# Estimation of Treatment Policy Estimands for Continuous Outcomes Using Off-Treatment Sequential Multiple Imputation

**DOI:** 10.1002/pst.2411

**Published:** 2024-08-04

**Authors:** Thomas Drury, Juan J. Abellan, Nicky Best, Ian R. White

**Affiliations:** 1 GSK, London, UK; 2 MRC Clinical Trials Unit at UCL, University College London, London, UK

**Keywords:** estimands, estimation, treatment policy, multiple imputation

## Abstract

The estimands framework outlined in ICH E9 (R1) describes the components needed to precisely define the effects to be estimated in clinical trials, which includes how post-baseline ‘intercurrent’ events (IEs) are to be handled. In late-stage clinical trials, it is common to handle IEs like ‘treatment discontinuation’ using the treatment policy strategy and target the treatment effect on outcomes regardless of treatment discontinuation. For continuous repeated measures, this type of effect is often estimated using all observed data before and after discontinuation using either a mixed model for repeated measures (MMRM) or multiple imputation (MI) to handle any missing data. In basic form, both these estimation methods ignore treatment discontinuation in the analysis and therefore may be biased if there are differences in patient outcomes after treatment discontinuation compared with patients still assigned to treatment, and missing data being more common for patients who have discontinued treatment. We therefore propose and evaluate a set of MI models that can accommodate differences between outcomes before and after treatment discontinuation. The models are evaluated in the context of planning a Phase 3 trial for a respiratory disease. We show that analyses ignoring treatment discontinuation can introduce substantial bias and can sometimes underestimate variability. We also show that some of the MI models proposed can successfully correct the bias, but inevitably lead to increases in variance. We conclude that some of the proposed MI models are preferable to the traditional analysis ignoring treatment discontinuation, but the precise choice of MI model will likely depend on the trial design, disease of interest and amount of observed and missing data following treatment discontinuation.

## 1 Introduction

The ICH E9 (R1) addendum, ‘Estimands and sensitivity analysis for clinical trials’, introduces the estimands framework and argues it should be used to precisely describe the effects to be estimated in clinical trials [1]. The framework defines the five components required for an estimand: a target population, an outcome variable, specified treatment conditions, a summary measure and a list of intercurrent events (IEs) with the strategies used to handle them. IEs are events that occur after initiation of treatment and impact the interpretation or existence of the primary outcome variable of interest. The guideline discusses several strategies to deal with IEs, including the treatment policy approach, which aims to assess the effect of treatment on outcomes for all patients regardless of the IE occurring.

A fundamental IE in most clinical trials is discontinuation from the protocol-assigned treatment. Adopting a treatment policy approach for this IE implies that outcomes after treatment discontinuation are relevant for the effect of interest. In this setting, patient discontinuation from treatment and patient withdrawal from the study (also referred to as withdrawal from follow-up) should be regarded as separate events, with the former being the IE and the latter creating missing data.

When a treatment policy strategy is chosen to deal with treatment discontinuation, patients should be encouraged to provide data after discontinuing from treatment until they complete follow-up. However, many patients are likely to withdraw from the study at the same time as they discontinue from treatment or at some intermediate time before the primary assessment, creating a missing data problem.

In this setting, it is typical for estimation methods to use all the observed outcome data pre- and post-discontinuation and rely on a basic missing at random (MAR) assumption. This assumption—which we will refer to as a *common MAR* assumption—states that any missing outcomes are comparable with observed outcomes from patients with similar outcome values at previous timepoints and similar baseline covariates, but who remained in the trial. Importantly, under this common MAR assumption, post-baseline factors other than outcome data until that timepoint are not used to inform about the similarity between patients.

In reality, this type of MAR assumption is likely to be implausible if the probability of missingness and the expected outcomes differ between the pre- and post-discontinuation data. When patients withdrawing from a study are no longer on protocol-assigned treatment, it may be more sensible to assume their missing data are more similar to observations collected after treatment discontinuation.

This paper has two aims. First, we propose a set of multiple imputation (MI) models for continuous repeated outcomes, which use observed pre- and post-discontinuation outcomes to impute missing post-discontinuation data, for use in trials with a continuous endpoint where treatment discontinuation is handled with a treatment policy strategy. The MI models are easy to implement in standard software and flexible, allowing for different behaviour before and after discontinuation. Second, we use a simulation study to assess the performance of the models for estimating the treatment effects in different scenarios. We compare results with the analysis of the full simulated data (i.e. without missing values), standard repeated measures analyses and other MI models. Based on the simulation study, we also make recommendations about which models may be more suitable in different situations.

MI methods that use the past data and treatment discontinuation status have been considered for recurrent-event data [2] and event time data [3]. Other MI models have also been proposed for continuous data [4–7]. Some of the models considered in this work are similar in structure to other implementations [4, 5], but other models we propose are more complex than previous work and investigate further potential estimation methods. The work is intended for study teams that are targeting treatment policy estimands for continuous endpoints to help them specify suitable models for imputing missing data when post-discontinuation outcomes are only partially collected. Example SAS code for all models is provided via GitHub (https://github.com/GSK-Biostatistics/mi_off_trt/tree/main/cont).

For clarity, this paper refers to patient outcomes before treatment discontinuation as ‘on-treatment’ and outcomes after treatment discontinuation as ‘off-treatment’. The label off-treatment is used because patients no longer receive the initially randomised treatment, but it is acknowledged that off-treatment patients could go on to receive rescue or alternative treatments as part of a clinical trial protocol.

The paper is organised as follows. Section 2 discusses the planning for a clinical trial which motivated this work, Section 3 specifies the methods including the imputation models, Section 4 outlines the characteristics of the simulation study with information on the data generation models, Section 5 details the results of the simulation study, Section 6 includes a discussion and finally Section 7 includes the conclusions we draw from this work.

## 2 Motivation

This work derives from the planning of a Phase 3 trial in patients with a respiratory disease to assess the potential for a new triple combination medicine to improve lung function over time. The trial planned to assess forced expiry volume in 1 s (FEV_1_) at repeated timepoints, and the patient outcome of interest was the change from baseline in FEV_1_ at the final timepoint. The design was a randomised parallel group trial with one group randomised to receive the triple combination and another group randomised to receive control. In the estimands framework, the trial planned to estimate:

Difference between Active and Control for mean change in FEV_1_ from baseline to the final timepoint for patients with the respiratory disease of interest regardless of treatment discontinuation.

Although the trial protocol planned to collect outcome data from patients who discontinued randomised treatment, it was expected that some patients would still withdraw from the study, and therefore the estimation strategy planned to use MI with on- and off-treatment outcomes to deal with the inevitable missing data problem. This created the question of how elaborate any MI model should be in order to accurately capture the on- and off-treatment behaviour when building a robust estimate of the intervention effects. This became the starting point for the assessment of the models in this work. The paper is written from the perspective of providing justification for the selection of analysis methods in a statistical analysis plan before the data are collected.

## 3 Methods

### 3.1 Notation

We consider the general setting of a two-group parallel trial, where *Z* denotes the randomised groups, with *Z* = *C* and *Z* = *A* denoting the groups of patients randomised to receive Control and Active, respectively. All notation applies to each individual in the trial and therefore no patient-level index is included. We define *Y*_*j*_ as the actual outcome value at the *j* th timepoint, *j* = 0, …, *J*, with 0 representing baseline and *J* the timepoint of the key assessment (for the motivating trial *J* = 3). We assume *Y*_0_ is always observed. Let *D*_*j*_ be a variable indicating whether the patient is still on randomised treatment (*D*_*j*_ = 0) or off-treatment (*D*_*j*_ = 1) at the *j*th timepoint. We assume a monotone discontinuation pattern and so any further timepoints are also off-treatment. If *D*_*J*_ = 0, the patient completes the trial on treatment. Also let *P*_*j*_ be a categorical variable representing the pattern of treatment discontinuation history up to the *j*th timepoint *j* = 0, …, *J*. As we assume that treatment discontinuation is monotone each *P*_*j*_ has *j* + 1 levels with *P*_*J*_ representing the *J* + 1 discontinuation patterns at the final timepoint.

### 3.2 Estimand

Applying the treatment policy strategy to treatment discontinuation in our motivating trial targets the estimand:

$${\text{Δ}}_{j}=E\left[Y_{j}-Y_{0}|Z=A\right]-E\left[Y_{j}-Y_{0}|Z=C\right],$$

which can be interpreted as the difference in expected change from baseline at timepoint *j* for patients randomised to receive Active compared with patients randomised to receive Control regardless of treatment discontinuations. The group-specific expected changes from baseline values on the right-hand side are also of interest where they provide further context to the treatment effects.

### 3.3 Estimation

With complete data, a standard estimation method for the quantity above would be a regression model for change from baseline (*Y*_*j*_ − *Y*_0_) with model terms for treatment group *Z* and baseline *Y*_0_. However, with incomplete data, it is usual to consider either mixed models for repeated measures (MMRM) or to use MI in a three-stage process: creating a set of complete data sets by imputing any missing outcomes, analysing the change from baseline *Y*_*j*_ − *Y*_0_ in each data set with the complete data model specified above, and combining the estimates using Rubin’s rules [8]. Both these estimation approaches are considered in this work and the models we use are specified below.

In the proposed MI models, outcomes for off-treatment patients may differ from on-treatment patients in mean and/or in variance. The core assumption underlying all the imputation models is that missing outcomes would behave like outcomes of different groupings of ‘similar’ patients who are still in the trial. For simplicity, the models are defined using pseudo-regression notation reflecting how they are specified programmatically. Full algebraic specifications for each model are available in Supporting Information S1. The interaction ‘star’ operator (*) between model terms indicates that separate terms are included for each level (or combination) of the categorical factors. In this notation, the model used for analysing complete data and imputed data, termed ANCOVA, is:

$$Y_{J}-Y_{0}=\;\text{Intercept}\;+Z+Y_{0}.$$

#### 3.3.1 MMRM

This analysis uses all the available data and makes no distinction between the on- and off-treatment outcomes and relies on the common MAR assumption. The model includes terms for treatment by timepoint and baseline by timepoint interactions and can be denoted as:

$$\begin{array}{l} Y=\;\text{Intercept}\;+Z+\;\text{Timepoint}\;+Y_{0}+Z*\;\text{Timepoint}\;\\ \;\;\;\;\;\;\;\;+Y_{0}*\;\text{Timepoint}.\; \end{array}$$

#### 3.3.2 Sequential MI Models Using Previous Outcomes

These proposed MI models are designed to be used sequentially for each timepoint *j* = 1, …, *J*, using previous outcomes, with separate parameters at each timepoint *j* and separate imputation for each treatment group. All models have a common intercept together with slope parameters on *Y*_0_, …, *Y*_*j*−1_ at each timepoint. Some models use indicators *D*_1_, …, *D*_*j*_ as covariates to distinguish between on- and off-treatment status and others use variables containing the pattern *P*_*j*_ of treatment discontinuation. Terms specified as *D*_*j*_ * *Y*_*j*_ and *P*_*j*_ * *Y*_*j*_ indicate separate *Y*_*j*_ covariate slope terms for each level of the categorical covariates.

##### Common Intercepts Common Slopes (CICS)

$$Y_{j}=\;\text{Intercept}\;+Y_{0}+\ldots +Y_{j-1}.$$

The CICS model is the simplest. It imputes missing outcomes for timepoint *j* based on a single intercept and single set of slopes for previous outcomes whether on- or off-treatment.

##### On/Off-Intercepts Common Slopes (OICS)

$$Y_{j}=\;\text{Intercept}\;+D_{j}+Y_{0}+Y_{1}+\ldots +Y_{j-1}.$$

The OICS model extends CICS by allowing separate intercept terms for on- and off-treatment outcomes when imputing missing values for timepoint *j*.

##### Pattern Intercepts Common Slopes (PICS)

$$Y_{j}=\;\text{Intercept}+P_{j}+Y_{0}+Y_{1}+\ldots +Y_{j-1}.$$

PICS extends OICS by including separate intercept terms for each treatment discontinuation pattern up to timepoint *j*.

##### On/Off-Intercepts On/Off-Slopes (OIOS)

$$Y_{j}=\;\text{Intercept}\;+D_{j}+Y_{0}+\ldots +Y_{j-1}+D_{j}*Y_{0}+\ldots +D_{j}*Y_{j-1}.$$

The OIOS model allows separate on- and off-treatment intercept and slope parameters. The slopes are separated according to the discontinuation status of the subject at the *current imputation timepoint j*. The discontinuation status of the current timepoint is used as it maintains the principle of ‘no interactions without main effects’.

##### Pattern Intercepts On/Off-Slopes (PIOS)

$$Y_{j}=\;\text{Intercept}+P_{j}+Y_{0}+Y_{1}+\ldots +Y_{j-1}+D_{0}*Y_{0}+\ldots +D_{j-1}*Y_{j-1}.$$

PIOS extends PICS. In addition to a separate intercept for each discontinuation pattern, it also allows the slopes to differ according to the discontinuation status of the patient at the time of the *previous outcomes*. As the pattern intercept term automatically includes each prior discontinuation status their inclusion for the slopes does not violate the ‘no interactions without main effects’ principle.

##### Pattern Intercepts Pattern Slopes (PIPS)

$$Y_{j}=\;\text{Intercept}+P_{J}+Y_{0}+Y_{1}+\ldots +Y_{j-1}+P_{J}*Y_{1}+\ldots +P_{J}*Y_{j-1}.$$

The PIPS model is the most flexible proposed and is structured so that all intercept and slope parameters are estimated with outcomes from patients with the same final treatment discontinuation pattern.

There are other potential imputation models or variations to the six proposed above. However, for this work, it was thought these six would cover a suitable range from very basic to very flexible imputation models. The idea was this range would allow conclusions to be drawn about the level of complexity required to achieve robust estimates given the likely data characteristics of the proposed trial. Table 1 summarises each model highlighting the similarities and differences.

#### 3.3.3 Sequential MI Using Previous Residuals

To compare with our proposed MI models, we also consider both models outlined in Roger [4]. These models condition on previous residual values $R_{j}=Y_{j}-{\hat{\mu }}_{j}$, where ${\hat{\mu }}_{j}$ is the estimated mean at the *j*th timepoint. Using the residuals to condition on previous timepoints leads to an expected value of zero for the residuals and therefore the remaining intercept parameters at each time-point coincide with marginal means from a multivariate normal.

##### On/Off-Intercepts With Common Slopes Using Residuals (OICS-R)

$$Y_{j}=\;\text{Intercept}+D_{j}+R_{0}+R_{1}+\ldots +R_{j-1}.$$

The OICS-R model adjusts the marginal means for on- and off-treatment, but makes no distinction between on- and off-treatment when conditioning on the residuals. This is similar in structure to the OICS model we propose, but it can be shown that these models are not equal (see Supporting Information S1).

##### Pattern Intercepts With Common Slopes Using Residuals (PICS-R)

$$Y_{j}=\;\text{Intercept}\;+P_{j}+R_{0}+R_{1}+\ldots +R_{j-1}$$

The PICS-R model adjusts the marginal means for each treatment discontinuation pattern possible up to timepoint *j*, but makes no distinction between on- and off-treatment when conditioning on the residuals. It can be shown that this model is a reparameterisation of the PICS model we propose (see Supporting Information S1).

## 4 Simulation Study

The goal of the simulation study was to assess the bias and precision of the estimated treatment effects from each model and to investigate the convergence and stability of the models. The design of the simulated trials matched the parallel group structure of the proposed Phase 3 trial that motivated this work. Each simulated trial included 375 patients per group and aimed to detect a clinically meaningful FEV_1_ difference of 100 mL between Active and Control.

**Figure 1:**
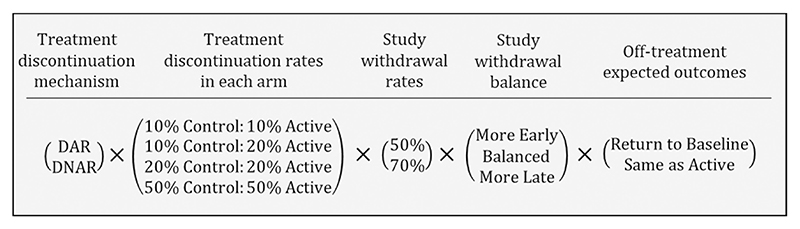
A schematic showing the full factorial nature of the simulation study with 96 Scenarios for each of the 1000 simulated studies.

For each of the simulated trials, we considered two treatment discontinuation mechanisms, four pairs of discontinuation rates, two study withdrawal rates, three withdrawal patterns and two off-treatment expected outcomes trajectories. This resulted in 96 discontinuation and withdrawal combinations with 1000 trials created for each (summarised in Figure 1). The large factorial design of the simulation was intended to ‘stress test’ the imputation models with respect to convergence and the accuracy of the imputations and estimates. The following parts of this section outline how the data were generated and provide detail on the different scenarios for treatment discontinuation, study withdrawal and off-treatment expected outcomes. The final part of this section gives information on the analyses and performance measures. Data generation, imputation and analysis were performed using SAS (SAS Institute Inc., Cary, NC, USA) and the OICS-R and PICS-R models were fitted using the MISTEP macro [9].

### 4.1 Data Generation

To create outcome data for virtual patients, we generated a vector of correlated patient-level data comprising potential on-treatment FEV_1_ data for all timepoints *and* potential off-treatment FEV_1_ data for all timepoints, using a multivariate normal distribution. At each post-baseline timepoint, some patients were selected to discontinue treatment based on the discontinuation mechanism and rate of the specific scenario (see Section 4.2), with subsequent values using the generated off-treatment data. Some of the discontinued subjects were then selected to be withdrawn from the trial according to the specific withdrawal for that timepoint (see Section 4.3) with further outcomes set to missing.

The expected values of FEV_1_ (in mL) for baseline and on-treatment outcomes in the Control group were set as [2140, 2470, 2520, 2540], and the expected values for the on-treatment effects (differences between Active and Control) were set to [0, 100, 100, 100] for Timepoints 0 (baseline), 1, 2 and 3, respectively. Two general scenarios were considered for the off-treatment expected values (see Section 4.4). Full details of the data generation process are included in Supporting Information S2.

### 4.2 Treatment Discontinuation Mechanism and Proportion

To investigate complex treatment discontinuation situations, two mechanisms were considered:

1. Discontinuation at random (DAR) based on lack of efficacy. This mechanism selected which patients discontinued after each timepoint using a propensity score linked to each patient’s previous on-treatment FEV_1_ value and selected the lowest (worst) ranked patients to discontinue.
2. Discontinuation not at random (DNAR) based on the lack of efficacy. This mechanism selected which patients to discontinue after each timepoint using a propensity score linked to each patient’s next potential on-treatment FEV_1_ value and selected the lowest (worst) ranked patients to discontinue.

For each treatment discontinuation mechanism above, four treatment discontinuation rates were considered for Control and Active groups corresponding to the percentage of patients that had discontinued treatment by the final timepoint. Three scenarios with equal discontinuation rates of 10%, 20% and 50% in Control and Active were created and one with unequal discontinuation rates of 10% Control and 20% Active. The values of 10% and 20% discontinuation were chosen as plausible rates for the planned clinical trial and the 50% value was included to understand a trial with an extreme level of treatment discontinuation.

The treatment discontinuations were spread over the post-baseline assessments *Y*_1_, *Y*_2_ and *Y*_3_ in the ratio 5:3:2, creating a larger proportion of patients discontinuing treatment at earlier timepoints. As an example, a rate of 50% treatment discontinuation would be allocated as (25%, 15%, and 10%) for Timepoints 1, 2 and 3, respectively.

### 4.3 Study Withdrawal Rate and Balance

The process for simulating which of the patients who discontinued treatment then withdraw from the trial (creating missing outcomes) was simplified to be missing completely at random (MCAR). However, given we specified that study withdrawal can only occur in patients that have discontinued treatment, the overall missing data process is conditional on the specific treatment discontinuation mechanism creating either MCAR|DAR or MCAR|DNAR processes. The former is an informative missing data process unless the observed discontinuation status is considered, and the latter is an informative missing data process even if the observed discontinuation is considered due to the DNAR process. The selection process was designed as a single assessment at the point of treatment discontinuation, where the patient either withdrew and provided no off-treatment data or remained in the trial providing off-treatment outcomes until completion. The proportion of discontinued patients simulated to withdraw from the study *by the final timepoint* was fixed at either 50% or 70%, but the balance of where the withdrawals occurred was varied across the post-baseline assessments with scenarios of:

1. ‘More early’ with a larger withdrawal rate for patients who discontinued at timepoint 1 and smaller withdrawal rates for patients who discontinued at Timepoints 2 and 3. This creates smaller proportions of observed off-treatment data relative to missing off-treatment data at the early timepoint, but larger proportions at later time points.
2. ‘Balanced’ withdrawal with the same withdrawal rate for all patients who discontinued, regardless of their time of discontinuing, creating the same proportions of missing off-treatment data at each timepoint.
3. ‘More late’ with a smaller withdrawal rate for patients who discontinued at Timepoint 1 and larger rates for patients who discontinued at Timepoints 2 and 3. This creates larger proportions of observed off-treatment data relative to missing off-treatment data at early timepoints, but smaller proportions at later timepoints.

Even though the amount of study withdrawal by the final timepoint was fixed, it should be noted that the ‘More early’ scenarios have more missing data across all visits due to the monotone structure of study withdrawal. The withdrawal scenarios were designed to investigate whether the proportion of observed and missing off-treatment data at different time-points impacts sequential imputation in terms of propagating bias and variance when estimating the treatment effects at the final timepoint.

### 4.4 Expected Values for Off-Treatment Outcomes

Two scenarios for the off-treatment outcomes were considered:

1. ‘Return to Baseline’. Under this assumption, patients who discontinue treatment would receive no further treatment and their mean FEV_1_ values would revert to baseline levels.
2. ‘Same as Active’. Based on the existing licensed medicines, it was considered possible for discontinuing patients to be offered further treatment using two separate components of the triple combination therapy being investigated, adhering to this alternative treatment correctly could result in similar FEV_1_ values compared with on-treatment patients receiving the active treatment.

### 4.5 Data Analysis

We analysed the full simulated on- and off-treatment data (before setting outcomes to missing) using the simple ANCOVA model defined in Section 3 for change from baseline FEV_1_ at the final timepoint with model terms for baseline FEV_1_ and treatment. Estimated marginal means (least-squared means in SAS terminology) were produced for the two arm-specific parts of the estimand. After setting generated post-withdrawal outcomes to missing, the MMRM analysis was fitted using restricted maximum likelihood with an unstructured variance− covariance matrix common to the two treatment arms. For the MI analyses, the imputation models were fitted each using 25 imputations for computational feasibility. The analysis model for each imputed data set was the same ANCOVA model used for the full data.

### 4.6 Performance Measures

The performance measures used for each model were the percentage of simulated data sets for which the model converged and produced stable estimates, and the bias, 95% confidence interval (CI) halfwidth and coverage of the estimated treatment effects (Active vs. Control) and the estimated group means. The bias and CI coverage were calculated using the analytical expected values (calculations included in Supporting Information S2). We also compared an estimate of Type 1 error under a null hypothesis of no difference in expected FEV_1_ values or discontinuation rates between each group. Monte Carlo (MC) errors were also calculated.

## 5 Results

### 5.1 Convergence and MC Error

There were no problems fitting the ANCOVA analysis on the full data or the MMRM model. All MI models except PIOS and PIPS (the most complex MI models) converged and produced stable estimates. The PIPS model had convergence issues in all scenarios with 10% treatment discontinuation and also scenarios with 20% treatment discontinuation and ‘More Early’ study withdrawal. The convergence issues were due to insufficient off-treatment data in some discontinuation patterns. In the remaining scenarios, the PIPS estimates had extreme variability and were not considered reliable. In general, it suggests the PIPS model could easily lead to problems if used in the planned respiratory trial and therefore this model was discarded. No further results or comparisons for PIPS are presented in this work.

The PIOS model had no convergence issues reported by the software, but for scenarios that combined 10% discontinuation rates, 70% withdrawal rates and ‘More Early’ withdrawal balance, the model estimates had extreme variance. The estimates from these scenarios were not considered reliable enough to include in comparisons in this work; however, the remaining scenarios for the PIOS model are presented.

For all other models and scenarios, the MC standard error for treatment effects were below 1.7 mL for the 50% withdrawal rates and below 2.2 mL for the 70% withdrawal rates (corresponding to 1.7% and 2.2% of the effect size, respectively).

### 5.2 Bias, Halfwidth and Coverage

**Figure 2:**
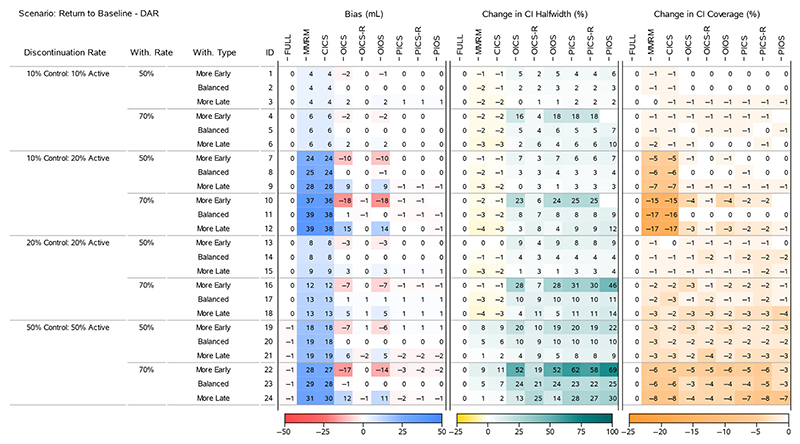
Simulation results for treatment effects in the Return to Baseline—DAR scenarios: the bias is calculated using analytical expected values, change in 95% CI halfwidth and coverage is relative to the 95% CIs for the full simulated data (FULL Results).

**Figure 3:**
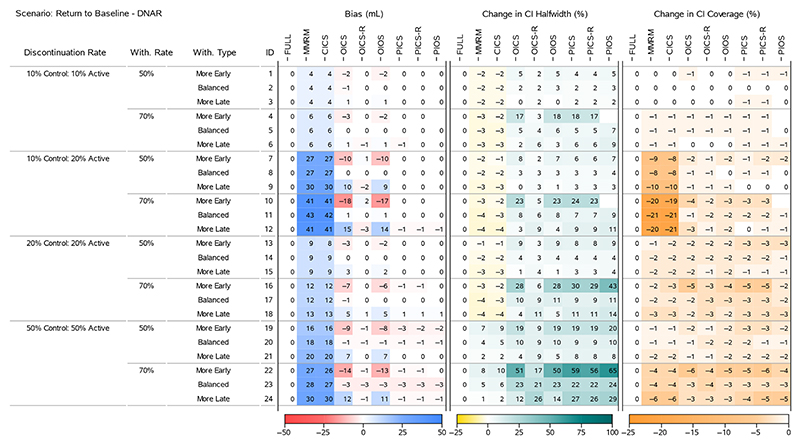
Simulation results for treatment effects in the Return to Baseline—DNAR scenarios: the bias is calculated using analytical expected values, change in 95% CI halfwidth and coverage is relative to the 95% CIs for the full simulated data (FULL Results).

**Figure 4:**
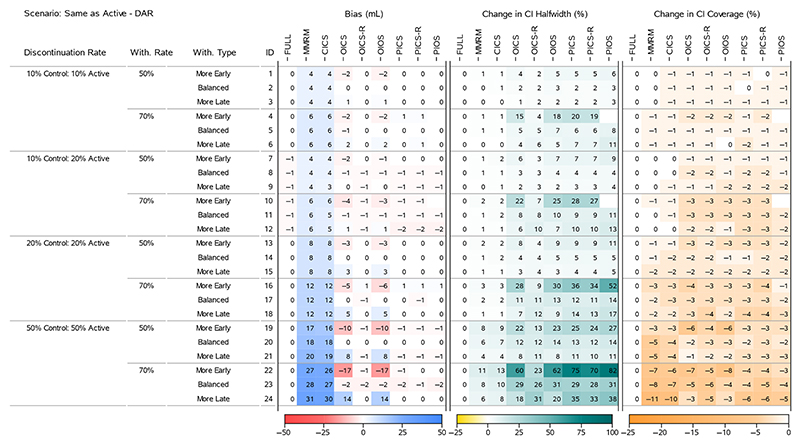
Simulation results for treatment effects in the Same as Active—DAR scenarios: the bias is calculated using analytical expected values, change in 95% CI halfwidth and coverage is relative to the 95% CIs for the full simulated data (FULL Results).

**Figure 5:**
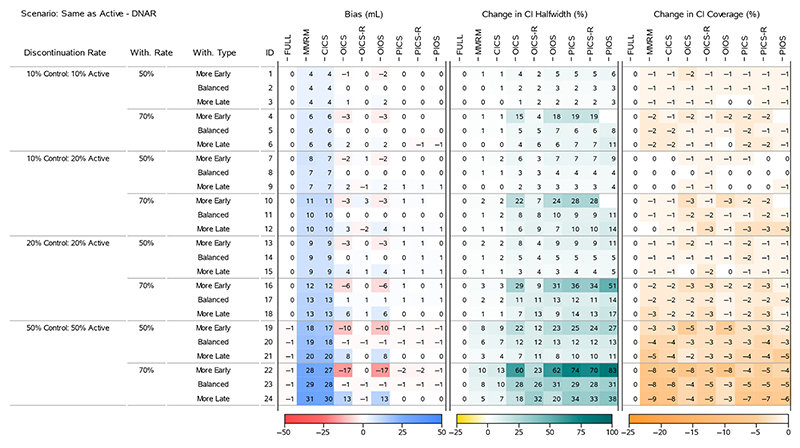
Simulation results for treatment effects in the Same as Active—DNAR scenarios: the bias is calculated using analytical expected values, change in 95% CI halfwidth and coverage is relative to the 95% CIs for the full simulated data (FULL Results).

To understand the results for the treatment effects across all scenarios, we present four heatmaps that summarise the bias, change in 95% CI halfwidth and change in 95% CI Coverage. Figures 2 and 4 display all the ‘Return to Baseline’ and ‘Same as Active’ scenarios for the DAR mechanism. Figures 3 and 5 display all the ‘Return to Baseline’ and ‘Same as Active’ scenarios for the DNAR mechanism. Detailed plots of the treatment effect bias, 95% CI halfwidth and coverage for each scenario are presented in Supporting Information S4−S6. Similar plots for the group means are available on the Github repository (https://github.com/GSK-Biostatistics/mi_off_trt/tree/main/cont). Any differences we discuss in the heatmaps can be observed in the Supporting Information figures and are not chance differences due to simulation (i.e. not due to MC error). We discuss the models in four categories—the full data ANCOVA (FULL), the models that rely on the common MAR assumption (MMRM and CICS), the models that have on- and off-treatment intercepts (OICS, OICS-R and OIOS) and the models with pattern-based intercepts (PICS, PICS-R and PIOS). We also consider similarities and differences between the corresponding scenarios for the two off-treatment expected outcome trajectories.

#### 5.2.1 Full Data ANCOVA Model

There was negligible bias for the treatment effects from the full data ANCOVA model which indicates that on average our simulated data matches the analytically calculated true expected values for the treatment policy in each scenario.

The CI halfwidths from the estimated treatment effects increased as the discontinuation rate increased, with larger increases seen in the ‘Return to Baseline’ scenarios compared with the corresponding ‘Same as Active’ scenarios (Figures in S4).

These provide a useful reference for comparing the other models against.

The full data analysis produced overcovered CI intervals for most scenarios (Figures 2 and 4). This is primarily due to the fixing of treatment discontinuation and study withdrawal in our DGM and correspond to the coverage for those exact discontinuation and withdrawal rates. We use these empirical coverage values for direct comparisons with the coverage from other models.

#### 5.2.2 Models With a Common MAR Assumption

The MMRM and CICS models produced biased treatment effects in all scenarios. The bias increased as the treatment discontinuation and withdrawal rates increased and was magnified further in scenarios with unequal discontinuation rates. The largest bias for the treatment effects for 50% and 70% study withdrawal was approximately 30 and 43 mL (corresponding to 30% and 43% of the target effect size), and occurred in the ‘Return to Baseline’ scenario with DNAR mechanism in combination with ‘10% Control 20% Active’ treatment discontinuation rates (Figure 3, IDs 9 and 11).

The bias in these models relates directly to the common MAR assumption—specifically that missing outcomes would be similar to an aggregate of all the observed on- and off-treatment outcomes at that timepoint. In all cases except for the patients in the Active group in the ‘Same as Active’ scenarios (not shown, but available on Github, https://github.com/GSK-Biostatistics/mi_off_trt/tree/main/cont), the missing off-treatment values are systematically different from the observed on-treatment values and not making a distinction results in MNAR data and bias.

The increase in bias across the discontinuation rates is driven by more discontinuations leading to more study withdrawal (which we fixed at 50% and 70% of treatment discontinuations) resulting in more MNAR data. Additionally, in the ‘Return to Baseline’ scenarios (which have large differences between on- and off-treatment), the treatment effect bias is compounded further by unbalanced treatment discontinuation which creates different amounts of MNAR induced bias for each group estimate (see Figures 2 and 4).

In some scenarios the MMRM and CICS models produced CI halfwidths for the treatment effects that were smaller than the full data. This occurred in the ‘Return to Baseline’ scenarios with 10% and 20% treatment discontinuation rates (Figures 2 and 3, IDs 1−18), but not for 50% treatment discontinuation rates nor any of the ‘Same as Active’ scenarios. The underestimation of variability also relates to the common MAR assumption that missing outcomes would be similar to all the observed on- and off-treatment data. In reality, the missing values were only similar to patients that had discontinued treatment. The scenarios that produced the underestimation in variability had quite different on- and off-treatment expected outcomes coupled with unequal discontinuation rates in the two arms, and not making a distinction between them implicitly assumes the data came from the whole range of observed values, rather than just the off-treatment values which then artificially reduces the overall variability estimates. This reduction appears to be outweighed or masked by the increased uncertainty due to larger numbers of study withdrawals seen in the cases with 50% treatment discontinuation and in general for the ‘Same as Active’ scenarios, where all off-treatment outcomes are similar to the on-treatment outcomes for patients in the Active group.

The CI coverage of the treatment effects for both the MMRM and CICS models were lower in all scenarios than the full data ANCOVA intervals. In general, the scenarios with the lowest coverages matched the situations with the largest bias implying the bias is the main cause for lack of coverage.

#### 5.2.3 Models With On- and Off-Treatment Intercepts

The OICS, OICS-R and OIOS models showed negligible bias for the treatment effects in all scenarios with ‘Balanced’ study withdrawal. However, in ‘More Early’ or ‘More Late’ study with-drawal scenarios, the OICS and OIOS models were biased with the direction linked to unbalancing. Again, the bias increased with discontinuation and withdrawal rate and was magnified further by unequal discontinuation rates and DNAR mechanism. In the scenarios with bias, the magnitude for OICS and OIOS was approximately the same with the maximum bias in both approximately 10 and 18 mL for 50% and 70% study with-drawal, respectively (corresponding to 10% and 18% of the target effect).

The OICS-R model also had biased treatment effects for scenarios with ‘More Early’ or ‘More Late’ study withdrawals, but only in the cases with unequal treatment discontinuation rates and DNAR mechanism (see Figure 3, IDs 10 and 12 and Figure 5, IDs 10, 12). The bias was consistently smaller than the OICS and OIOS models with a maximum absolute bias of 3 mL (3% of the target effect).

The unbalanced study withdrawal complicates the missing data mechanism making it depend on the time of treatment discontinuation. This implies the missingness is also related to the pattern of treatment discontinuation and explains why we see bias in OICS, OICS-R and OIOS models in these scenarios as they have no pattern components.

The CI halfwidths for the treatment effects in the OICS, OIOS and OICS-R models were similar to or greater than the full data ANCOVA, MMRM and CICS models in all scenarios. All three models had similar CI halfwidths for scenarios with ‘Balanced’ study withdrawal, but for ‘More Early’ study withdrawal the OICS and OIOS halfwidths were larger than the OICS-R and for ‘More Late’ withdrawal they were lower than OICS-R. The maximum percentage change in halfwidths relative to the FULL model for the OICS, OICS-R and OIOS models were 23%, 13% and 23%, respectively, for 50% study withdrawal (Figure 4, ID 19), and maximums of 60%, 32% and 62% for 70% study withdrawal (Figure 5, IDs 22 and 24).

The CI coverage for the treatment effects were largely similar between the OICS, OICS-R and OIOS models. For 50% withdrawal, the coverage was similar to the full data ANCOVA except in the ‘Same as Active’ scenarios with 50% discontinuation rate and ‘More Early’ study withdrawal, where there was drop in coverage in all three models. For 70% withdrawal, there was a drop in coverage for ‘10% Control 20% Active’ discontinuation and the 50% discontinuation scenarios. Although not significantly lower, the point estimate for the drop in coverage for the OICS and OIOS models were consistently lower compared with the OICS-R model. The lowest coverage for 50% and 70% withdrawal was approximately 88% and correspond to a percentage change of 8% in the OICS and OIOS models.

Comparing the performance of the OICS and OICS-R models, it seems that the use of the residuals in the OICS-R model leads to smaller bias in all cases. The maximum bias for the treatment effects was also considerably smaller (16 mL vs. 4 mL). The use of the residuals also appears to limit the increase in variability considerably in the majority of scenarios (but not all). The maximum change in CI halfwidth was also considerably smaller (60% vs. 32%). The OICS-R model also appears to be unbiased in some situations with equal discontinuation rates where the missingness is related to discontinuation pattern and we see bias in OICS.

Comparing the structure of both (see S1), it is clear that the use of residuals for conditioning in OICS-R implies that the regression terms account for the on- or off-treatment status of the past values. This is not the case for the OICS model where the direct conditioning on the values makes no distinction between the status of the past data. To illustrate this, consider a patient that discontinued after Timepoint 0. Their slope estimation for Timepoint 1 would be the same for OICS-R and OICS, but at Timepoint 2 the slope estimation for OICS-R would be in reference to the mean for off-treatment outcomes at Timepoint 1, whereas the slope estimation for OICS would be in reference to the mean from all the on- and off-treatment outcomes at Timepoint 1. This difference implies the OICS-R has more structural flexibility to condition on the past history and may explain why the model outperforms the simpler OICS model in these scenarios. It should be noted that although OICS-R was unbiased for treatment effects in these cases, the group means were biased (not shown, but available on the Github repository, https://github.com/GSK-Biostatistics/mi_off_trt/tree/main/cont). However, with approximately the same treatment discontinuation in both groups and fixed study withdrawal, the difference between the groups is maintained. If there was unequal discontinuation or withdrawal rates between groups, this model is also likely to show bias.

#### 5.2.4 Models With Pattern-Based Intercepts

There was negligible bias for the treatment effects in the PICS, PICS-R and PIOS models in all scenarios. The lack of bias for the simpler pattern models (PICS and PICS-R) suggests the inclusion of pattern-based intercepts is sufficient to deal with the most complex scenarios we generated including time dependent missingness from unbalanced study withdrawal and DNAR mechanisms. One reason for this sufficiency is likely to be that our data generation model used the same variance−covariance values for on-treatment and off-treatment outcomes. If we had created data with different variances for on- and off-treatment, the PIOS model may have performed better the PICS and PICS-R.

For 50% study withdrawal, all three models had similar CI half-widths except scenarios with ‘More Early’ study withdrawal where the PIOS model halfwidths were slightly higher. The largest change in halfwidth occurred in the ‘Same as Active’ scenario with DAR mechanism, 50% discontinuation and ‘More Early’ withdrawal and was approximately 27% (Figure 3, ID 19). For 70% study withdrawal, there were consistent differences between the halfwidths of each model in the ‘More Early’ scenarios. The PIOS model had the greatest increase with the PICS showing slightly larger increases than the PICS-R model. The maximum increases for the PICS, PICS-R and PIOS models were 75%, 70% and 83%, respectively, and all occurred in the ‘Same as Active’ scenarios.

The increase in variability seen in PICS, PICS-R and PIOS is driven by the increased model complexity. The use of parameters to distinguish between discontinuation patterns results in a considerable reduction of information contributing to the estimation of each parameter and is the main driver behind the substantial increases to the variability.

The CI coverage for the treatment effects were largely comparable between the PICS, PICS-R and PIOS models. For 50% withdrawal, the coverage was similar to the full data ANCOVA except in the ‘Same as Active’ scenarios with 50% discontinuation rate and ‘More Early’ study withdrawal, where there was drop in coverage in all three models. For 70% withdrawal, there was a drop in coverage for all the 50% discontinuation scenarios. The lowest coverage for 50% and 70% withdrawal was approximately 91% and 90%, respectively, which corresponds to a percentage change of 4% and 8% compared with the coverage of the full ANCOVA estimates.

Finally, the bias, halfwidth and coverage results for PICS and PICS-R also provides confirmation by simulation that these models are essentially the same in structure, but for large amounts of treatment discontinuation and extreme levels of study withdrawal, the PICS-R model structure appears slightly more efficient.

#### 5.2.5 Comparing Off-Treatment Scenarios

Comparing the models that showed bias (MMRM, CICS, OICS, OIOS and OICS-R) for each ‘Return to Baseline’ and corresponding ‘Same as Active’ scenarios, we see similar levels of bias in the treatment effects when the treatment discontinuation rates are equal, but unequal discontinuation creates much larger bias for the ‘Return to Baseline’ scenarios. Looking at the bias for the corresponding group means for each of these cases (included on Github, https://github.com/GSK-Biostatistics/mi_off_trt/tree/main/cont) shows that there is larger bias in all the ‘Return to Baseline’ scenarios compared with the corresponding ‘Same as Active’. This difference is largely driven by the fact the ‘Return to Baseline’ off-treatment data are different to both Control and Active on-treatment data, whereas the ‘Same as Active’ scenario has off-treatment data matching the on-treatment data in the Active group and therefore the common MAR assumption holds in this group. The similar bias seen in the treatment effects for equal treatment discontinuation rates is driven largely by the choice of our simulated data. The bias in the group means and treatment effects in these scenarios are considered in more detail in Supporting Information S3.

### 5.3 Type 1 Error Control

The results for Type 1 error as a percentage are shown in Supporting Information S4. The tables show the estimated (two-sided) Type 1 error for each model under a null hypothesis of no difference between each groups expected outcomes while on- or off-treatment. With 1000 simulations, the MC error was approximately 1% and therefore any Type 1 error estimates of 7% or greater have been highlighted in red.

There were no Type 1 error inflations for the FULL data ANCOVA or the models relying on the common MAR assumption (MMRM and CICS).

For discontinuation rates of 10% and 20%, there were no Type 1 error inflation issues scenarios with 50% study withdrawal except the ‘Same as Active’ DNAR scenarios with ‘More Early’ study withdrawal.

For 50% treatment discontinuation and many cases with 70% withdrawal, there was Type 1 error inflation for all the on/off and pattern intercept models in at least one of the scenarios. The maximum Type 1 error was approximately 11% in the pattern-based models (PICS, PICS-R and PIOS), occurring in the Same as Active scenario with 50% treatment discontinuation and 70% ‘More Early’ withdrawal. Investigating these increases suggest they are driven by an underestimation of the model based standard errors which are systematically lower than the standard deviation of the point estimates simulated (results not shown, but available on Github, https://github.com/GSK-Biostatistics/mi_off_trt/tree/main/cont).

## 6 Discussion

This work assessed a number of different sequential MI models for the estimation of treatment effects where the IE of treatment discontinuation is handled using the treatment policy strategy. The inspiration came from Phase 3 design work for a respiratory drug where there were concerns about the accuracy of simple MMRM or MI models due to their reliance on a *common* MAR assumption across on- and off-treatment outcomes. We suggested a range of MI models to investigate the accuracy of performing imputation conditioning on previous values and treatment-discontinuation status or pattern. We evaluated the models for feasibility as estimation methods by comparing them with an ANCOVA analysis of the full data and a traditional MMRM using all available data.

The problems faced in fitting the PIPS model suggest it may have limited utility in many real trial applications. It may still be useful for large trials, but the ease of fitting and good performance of other MI models considered here suggests there is limited need for that level of complexity in the situations we explored.

Our work shows that accurate estimation is possible with some of the models we investigated in the majority of plausible scenarios, but in cases with large discontinuation rates and/or large withdrawal rates, the best performing models may still underestimate variability and have Type 1 error concerns. Therefore, if these models are to be used for estimation of primary estimands in real clinical trials, further work would be advisable to check the Type 1 error and efficiency at the design stage for the specific study using the expected discontinuation and withdrawal rates.

In addition, although we stress-tested the MI models by creating scenarios with small amounts of observed off-treatment data at particular timepoints, we only considered a simplified withdrawal set-up that ensured off-treatment outcomes existed for any situation where missing data needed imputing. In reality, this may not be the case for all trials and further work could look at how likely these models are to run into fitting problems if patients can leave the trial at any timepoint, as this may lead to smaller amounts of off-treatment data collected and consequently the imputation models becoming non-estimable. We also ensured any discontinuation and withdrawal was monotone, a simplification that is unlikely to occur in practice. Although this is not a major limitation, it can make sequential MI more difficult because sequential imputation of missing data at a given visit does not take into account the observed data in future visits. Further work to investigate the impact of intermittent missingness on sequential MI and also comparing it with multivariate imputation would be interesting.

A major point for consideration is that failure to collect data off-treatment may have a serious impact on reliable estimation of the estimand targeted here (regardless of the estimation method considered). Our simulation results show that the uncertainly in the group means and treatment effects increases considerably with increasing amounts of missing off-treatment data. Investigating this further (Supporting Information S3) suggests that the relationship depends on the overall amount of missing off-treatment data *and* the relative proportion of observed and missing off-treatment data. It is possible that a change of mindset and educational work is needed to increase awareness among study investigators and participants about the importance of collecting data after discontinuation of treatment.

In an extreme case where little to no off-treatment data were collected, the only feasible models considered in this work would be the common MAR-based methods; however, the resulting estimates and effects would really be based on data collected on-treatment. Such estimates would be more aligned with an estimand that uses a hypothetical strategy to deal with treatment discontinuation. Another option could be reference-based imputation methods [6, 10]. This alternative also targets a treatment policy estimand and would be appropriate in settings where subjects in the Active treatment arm who discontinue treatment receive post-discontinuation medication comparable with that in the Control arm. A detailed comparison of off-treatment MI with reference-based MI would also be an interesting extension to this work.

Another implication from these results is that a major factor in the size of bias when estimating a treatment policy estimand is the difference between on- and off-treatment outcomes. The large biases seen in our ‘Return to baseline’ scenarios result from baseline measures that were very different to the outcome behaviour of both the Active- and Control-treated subjects. Although this extreme case is plausible in some trials that use an Active comparator or expect some placebo effect, a situation where Control and off-treatment outcomes are similar may be more common—this type of data structure is sometimes described as a ‘jump to reference’ setting. The data generated in our ‘Same as Active’ scenarios are mirror images of this and therefore we would expect bias to be of similar magnitude for the corresponding treatment discontinuation and study withdrawal rates.

## 7 Conclusions

In general, this work has demonstrated that treatment discontinuation complicates the missing data mechanism associated with study withdrawal and can make the common MAR assumption invalid. It also demonstrated that imbalances of treatment discontinuation rates across the treatment groups, the size and imbalance of study withdrawal rates across time, DNAR mechanisms as well as different outcome behaviour on- and off-treatment will all create problems for estimating the effects of a treatment policy. In real trials, the majority of missing outcomes will be from patients discontinuing treatment and then withdrawing from the study. It is also likely that the discontinuation rates (and therefore the missing rates) will differ between the trial groups as larger numbers may discontinue from Control groups for lack of efficacy or larger numbers may discontinue from Active groups for tolerability issues. The time of discontinuation is also likely to affect whether a patient remains in the trial until the final timepoint—for example, it seems unlikely that patient discontinuing treatment 2 months into a 2-year trial will have the same chance of being observed at the final time-point compared with a patient discontinuing treatment 1 month before the trial ends. Given these likely scenarios, estimating an estimand with treatment policy handling of treatment discontinuation needs careful thought and is unlikely to be straight-forward in many cases.

No model we considered for estimating these effects seems to be optimal in all the scenarios we investigated. Given the large biases seen in the common-MAR-based (MMRM and CICS) models when there are only moderate amounts of unbalanced treatment discontinuation, we conclude these are poor choices for estimating treatment policy-based effects. An exception to this could be when the behaviour on- and off-treatment is expected to be similar or when there are low numbers of discontinuations and/or little missing data. The proposed alternative sequential imputation models that distinguish between on- and off-treatment data are clearly better and generally perform well for moderate rates of discontinuation and withdrawal, but involve a trade-off between bias and variability. The pattern-based intercept models (PICS and PICS-R) appear to be the best choice for reducing bias, but in some settings lead to sizable increases in variability which will clearly impact power. They also have the potential for estimation problems in real trials where lack of off-treatment data may make specific discontinuation patterns inestimable. It is clear from the on- and off-treatment intercept models OICS, OICS-R and OIOS that regression on the residuals is optimal and the OICS-R model can offer a good compromise by conceding small levels of bias in some settings as a trade-off for much less variance inflation. Logically, as it has fewer parameters than the pattern-based models it is also less likely to suffer from estimation issues related to lack of collected off-treatment data.

A pragmatic estimation strategy could be the pre-specification of a hierarchy of these models based on criteria related to successful fitting. As an example, the hierarchy could start with a pattern-based MI approach such as PICS or PICS-R, and if this failed the acceptance criteria then an intercept model such as OICS-R could be used. Finally, as a last resort if all off-treatment models failed the acceptance criteria, the hierarchy could default back to the CICS or MMRM approaches.

## Supplementary Material

Supporting Information

## Figures and Tables

**Table 1:** Sequential MI models compared.

		Slopes		
		Common	On/off	Pattern
Intercept	Common	CICS	—	—
	On/Off	OICS	OIOS	—
	Pattern	PICS	PIOS	PIPS

## Data Availability

Data sharing is not applicable to this article as no new data were created or analyzed in this study.
