# Supplementary material for: Estimation of Treatment Policy Estimands for Continuous Outcomes Using Off-Treatment Sequential Multiple Imputation
Source: Pharm Stat. Author manuscript; Available in PMC 2025 Jan 9. (PMC11602932; doi:10.1002/pst.2411)
Supplement: Supporting Information [file EMS201287-supplement-Supporting_Information.pdf]

Supporting Information

Additional supporting information can be found online in the Supporting Information section.

15391612, 0, Downloaded from https://onlinelibrary.wiley.com/doi/10.1002/pst.2411 by University College London UCL Library Services, Wiley Online Library on [27/11/2024]. See the Terms and Conditions (https://onlinelibrary.wiley.com/terms-and-conditions) on Wiley Online Library for rules of use; OA articles are governed by the applicable Creative Commons License
