# Supplementary material for: Estimation of Treatment Policy Estimands for Continuous Outcomes Using Off‐Treatment Sequential Multiple Imputation
Source: Pharm Stat. 2024 Aug 4;23(6):1144–55. doi: 10.1002/pst.2411 (PMC11602932; doi:10.1002/pst.2411)

SI Figure 2.1
Average 95% CI Halfwidth for Treatment Effects (Active vs. Control)

Disc. Scenario: Return To Baseline
Disc. Mechanism: DAR
With. Rate: 50%

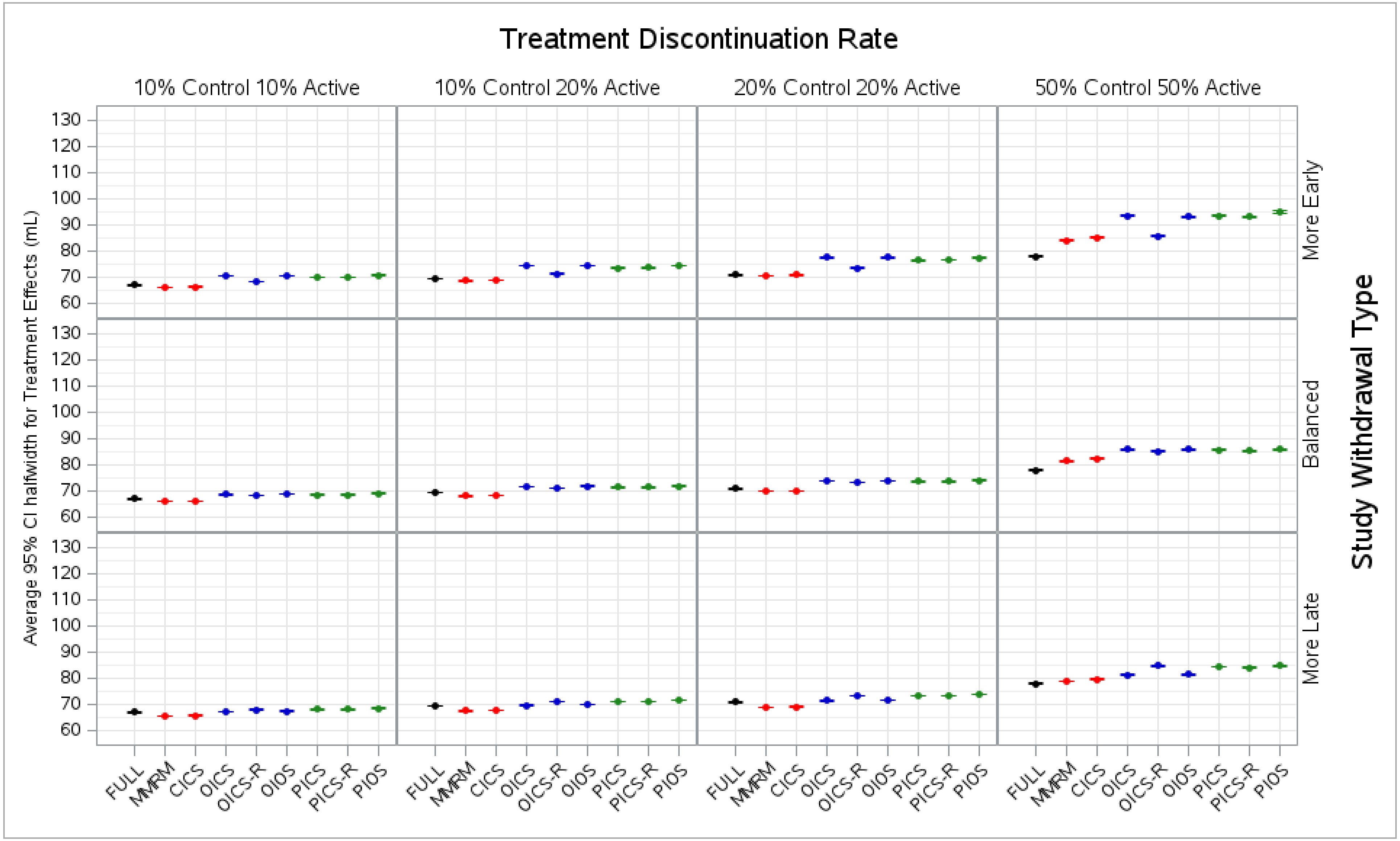

SI Figure 2.2
Average 95% CI Halfwidth for Treatment Effects (Active vs. Control)

Disc. Scenario: Return To Baseline
Disc. Mechanism: DAR
With. Rate: 70%

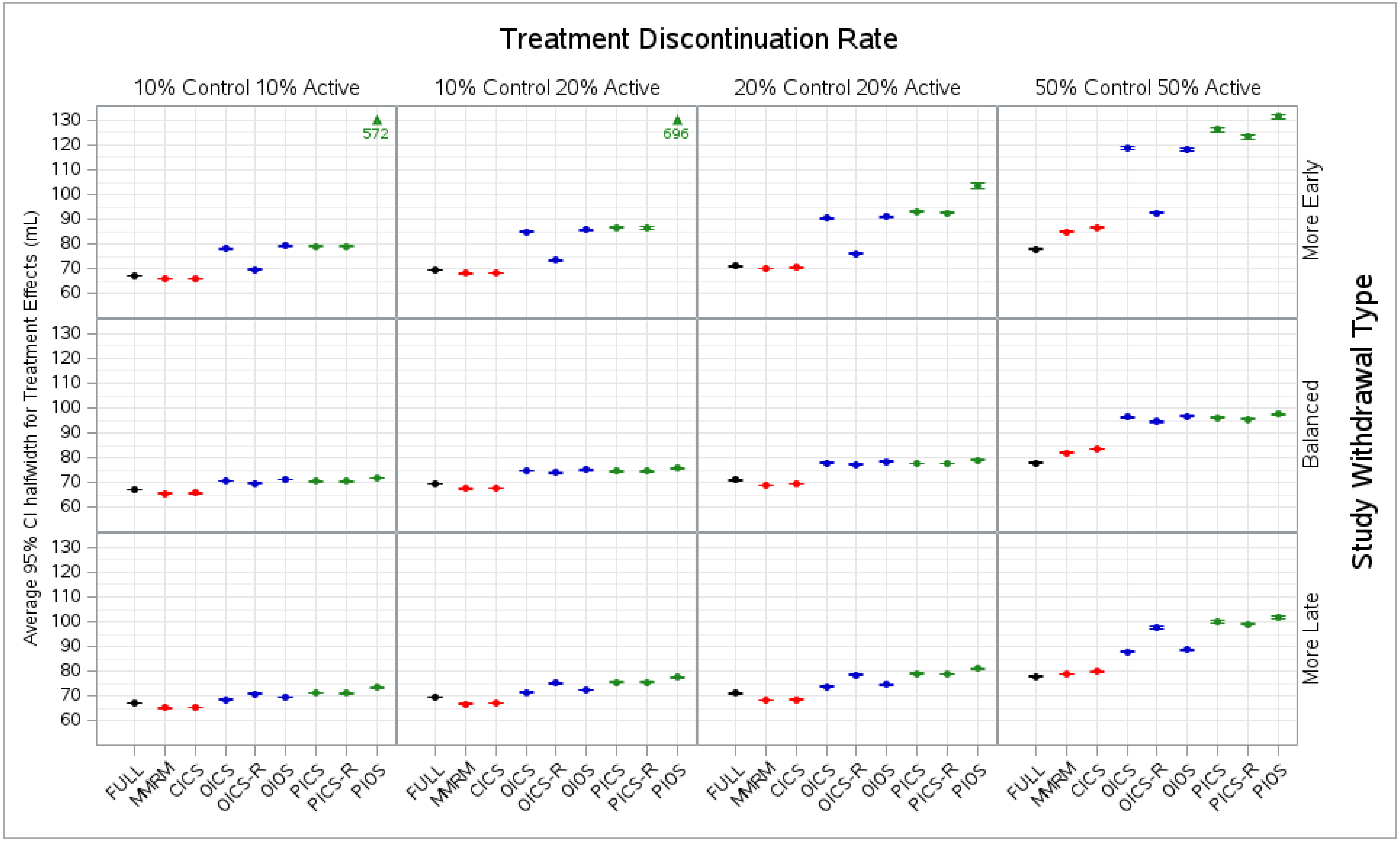

SI Figure 2.3
Average 95% CI Halfwidth for Treatment Effects (Active vs. Control)

Disc. Scenario: Same As Active
Disc. Mechanism: DAR
With. Rate: 50%

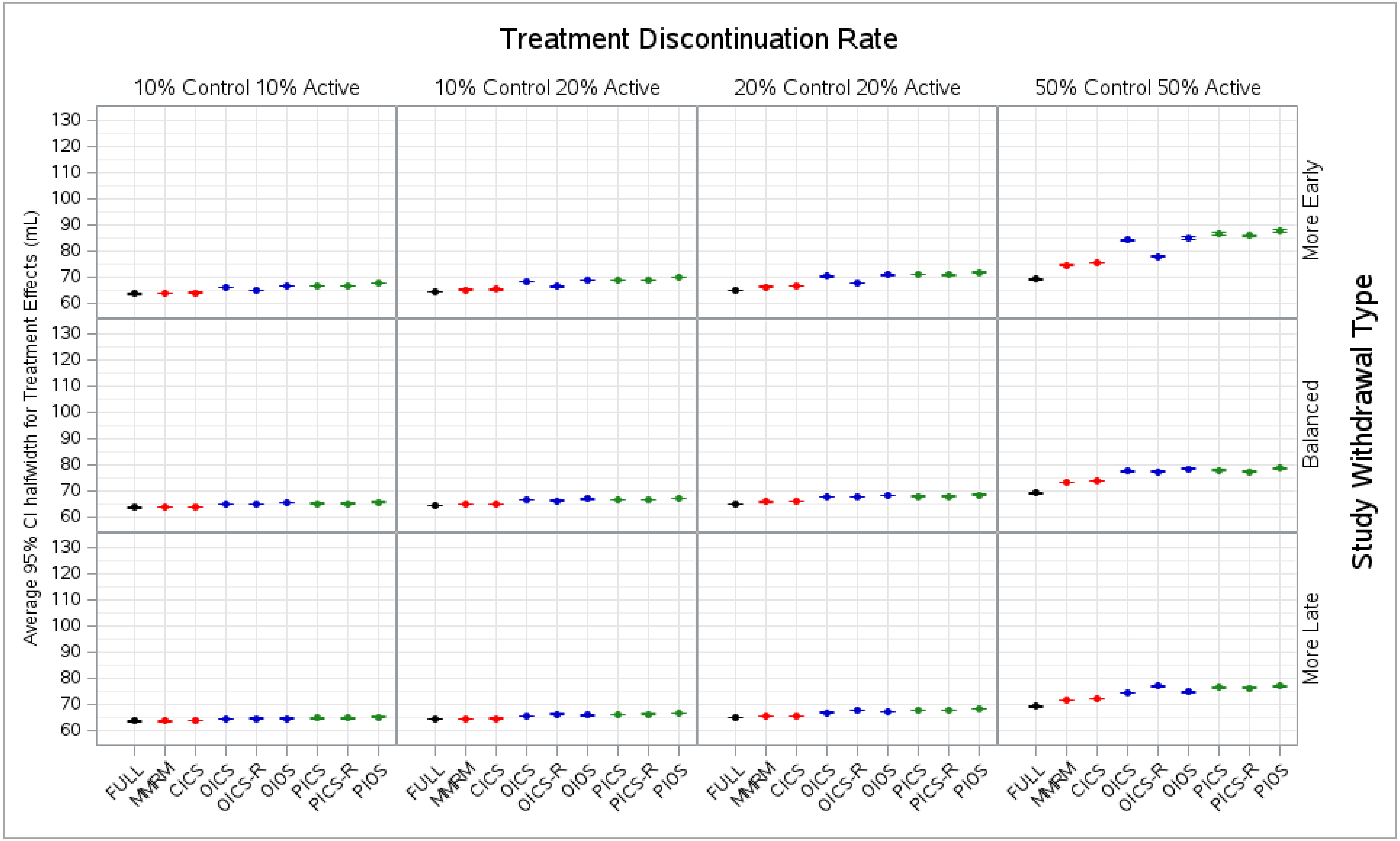

SI Figure 2.4
Average 95% CI Halfwidth for Treatment Effects (Active vs. Control)

Disc. Scenario: Same As Active
Disc. Mechanism: DAR
With. Rate: 70%

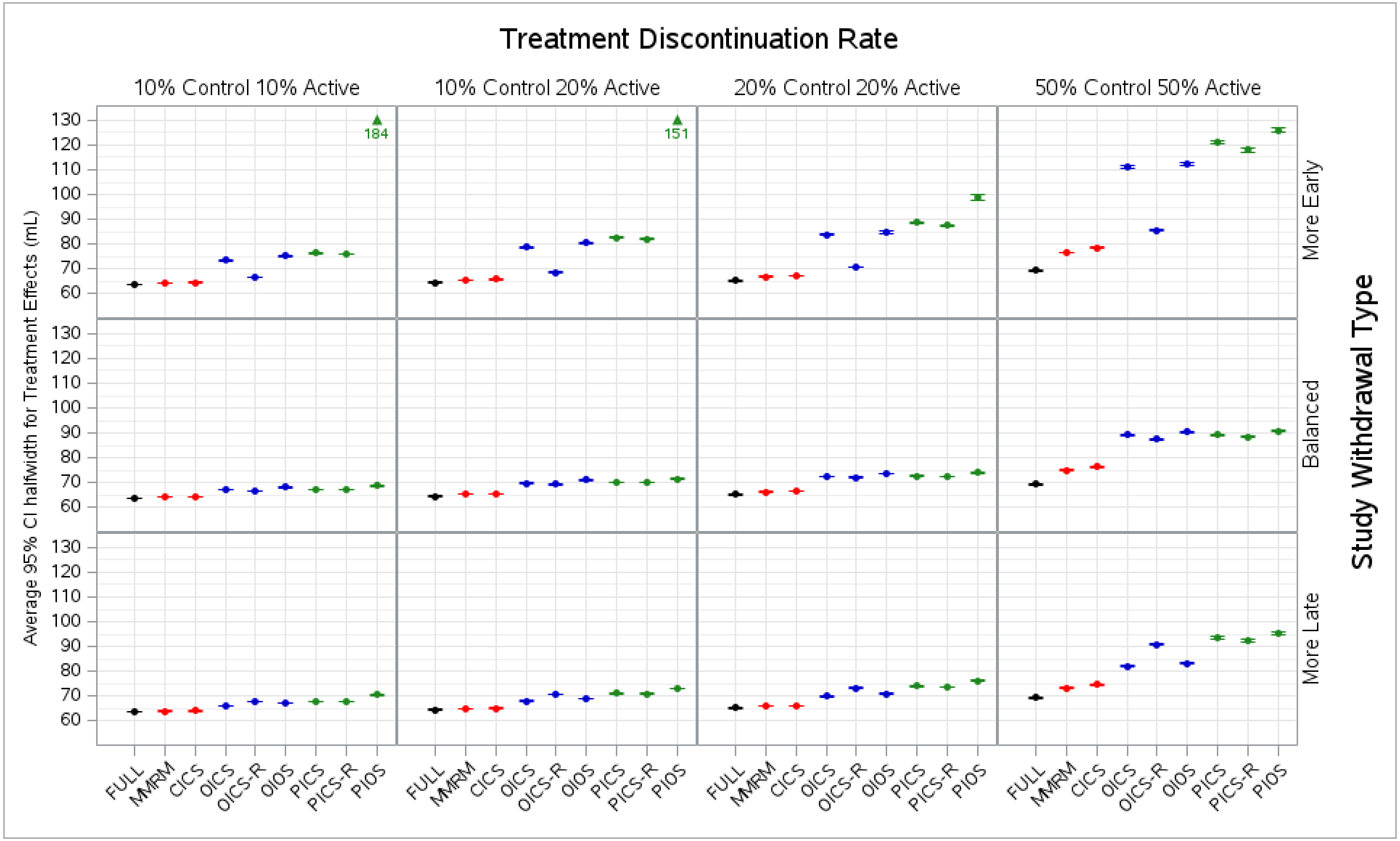

SI Figure 2.5
Average 95% CI Halfwidth for Treatment Effects (Active vs. Control)

Disc. Scenario: Return To Baseline
Disc. Mechanism: DNAR
With. Rate: 50%

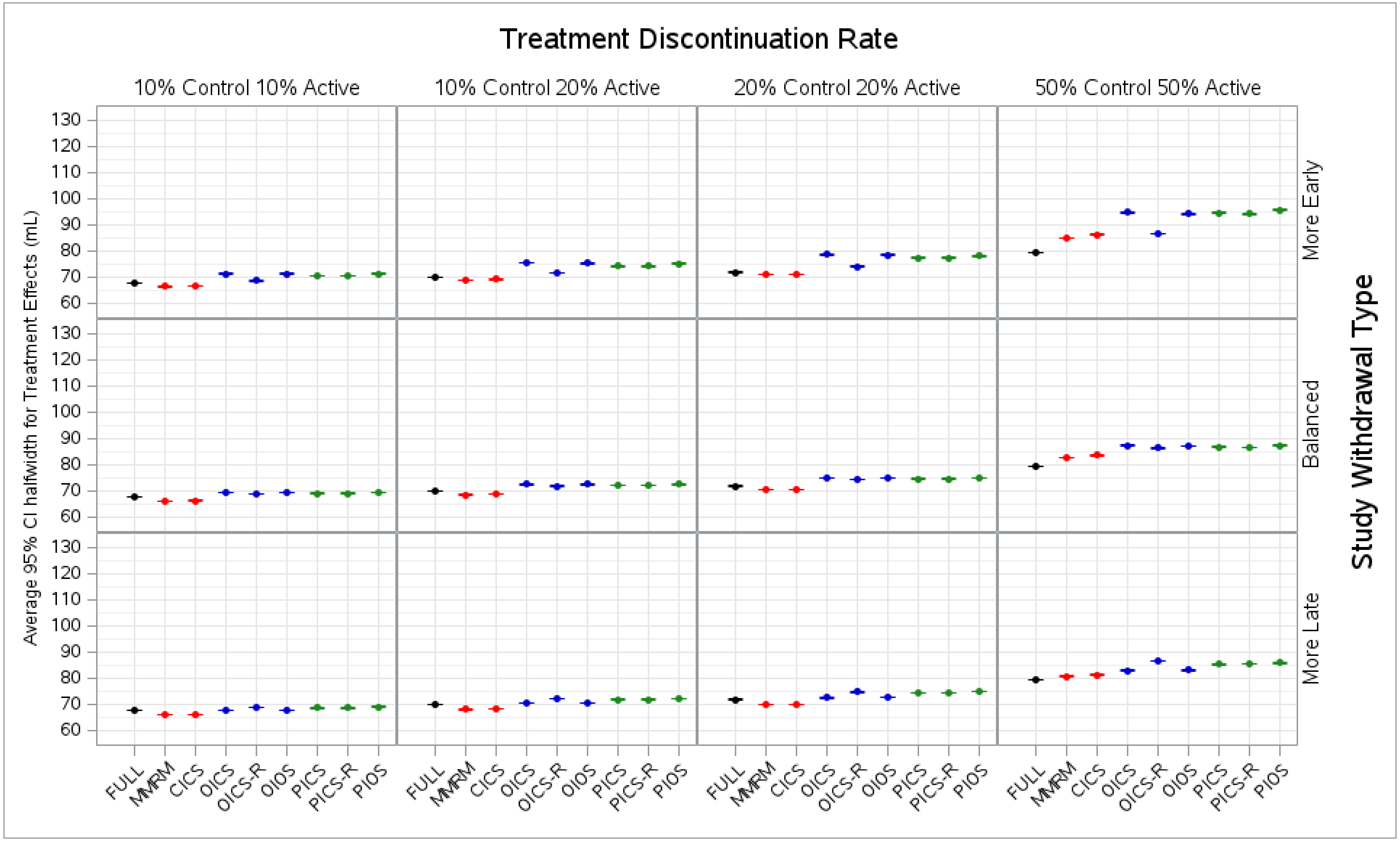

SI Figure 2.6
Average 95% CI Halfwidth for Treatment Effects (Active vs. Control)

Disc. Scenario: Return To Baseline
Disc. Mechanism: DNAR
With. Rate: 70%

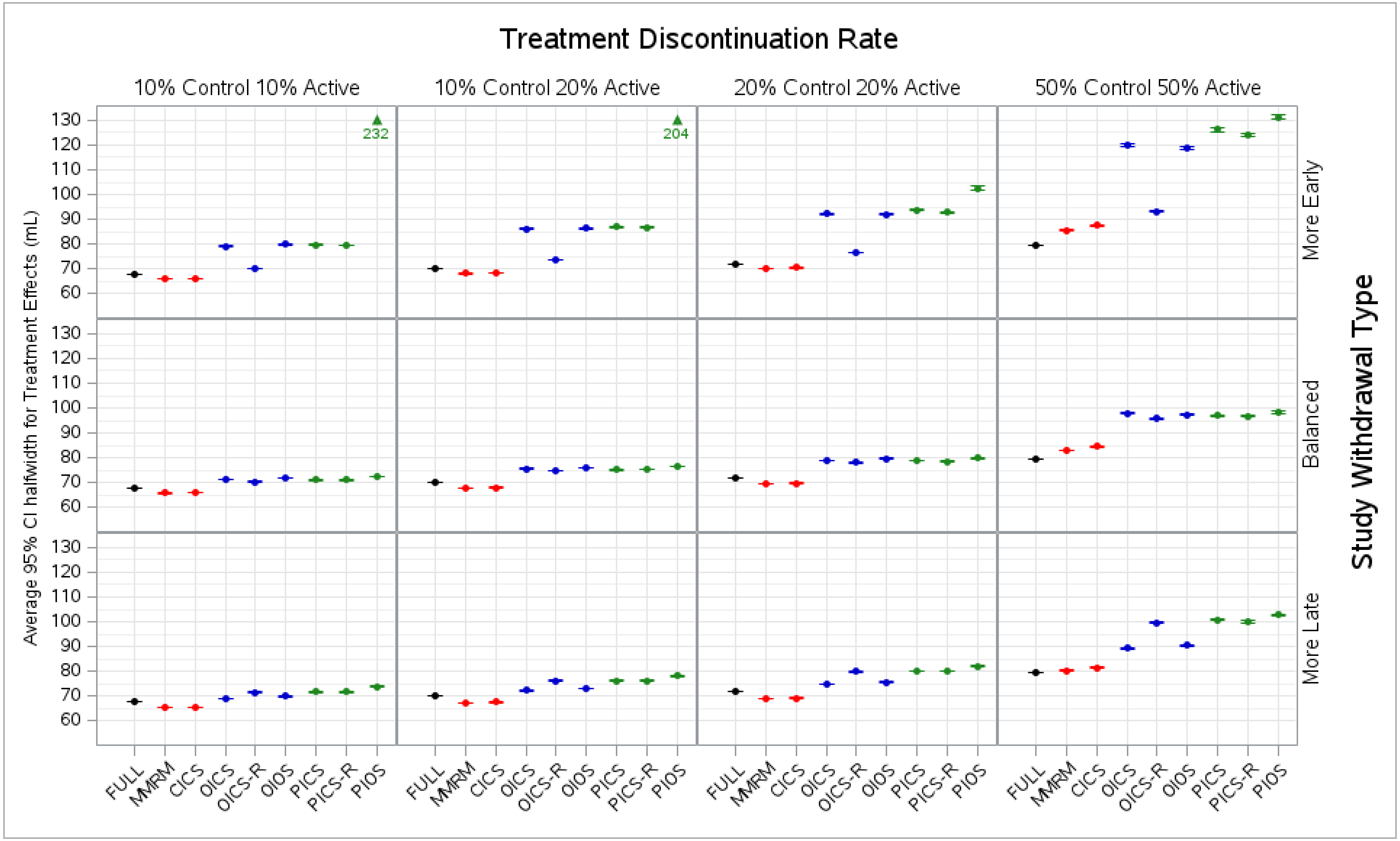

SI Figure 2.7
Average 95% CI Halfwidth for Treatment Effects (Active vs. Control)

Disc. Scenario: Same As Active
Disc. Mechanism: DNAR
With. Rate: 50%

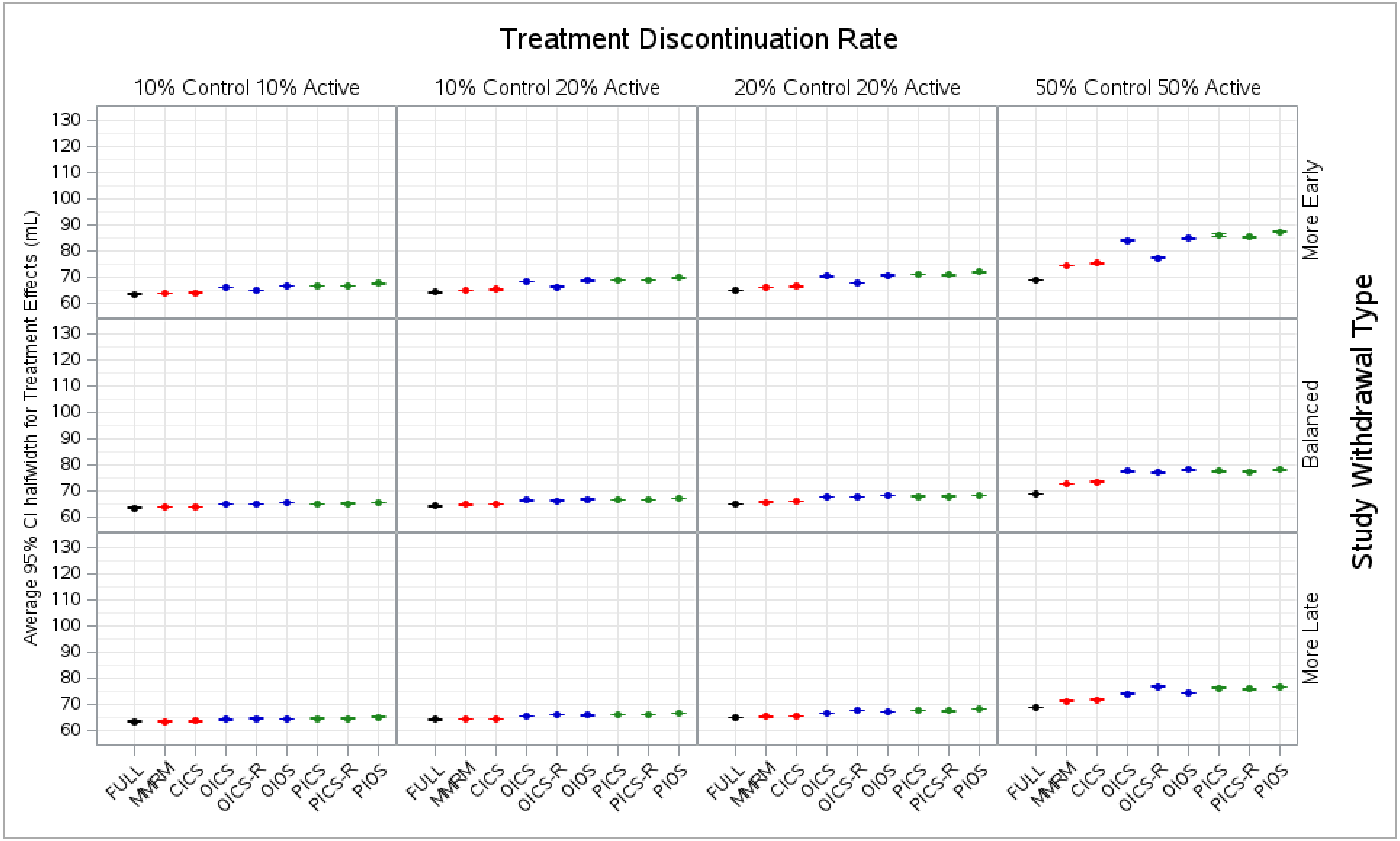

SI Figure 2.8
Average 95% CI Halfwidth for Treatment Effects (Active vs. Control)

Disc. Scenario: Same As Active
Disc. Mechanism: DNAR
With. Rate: 70%

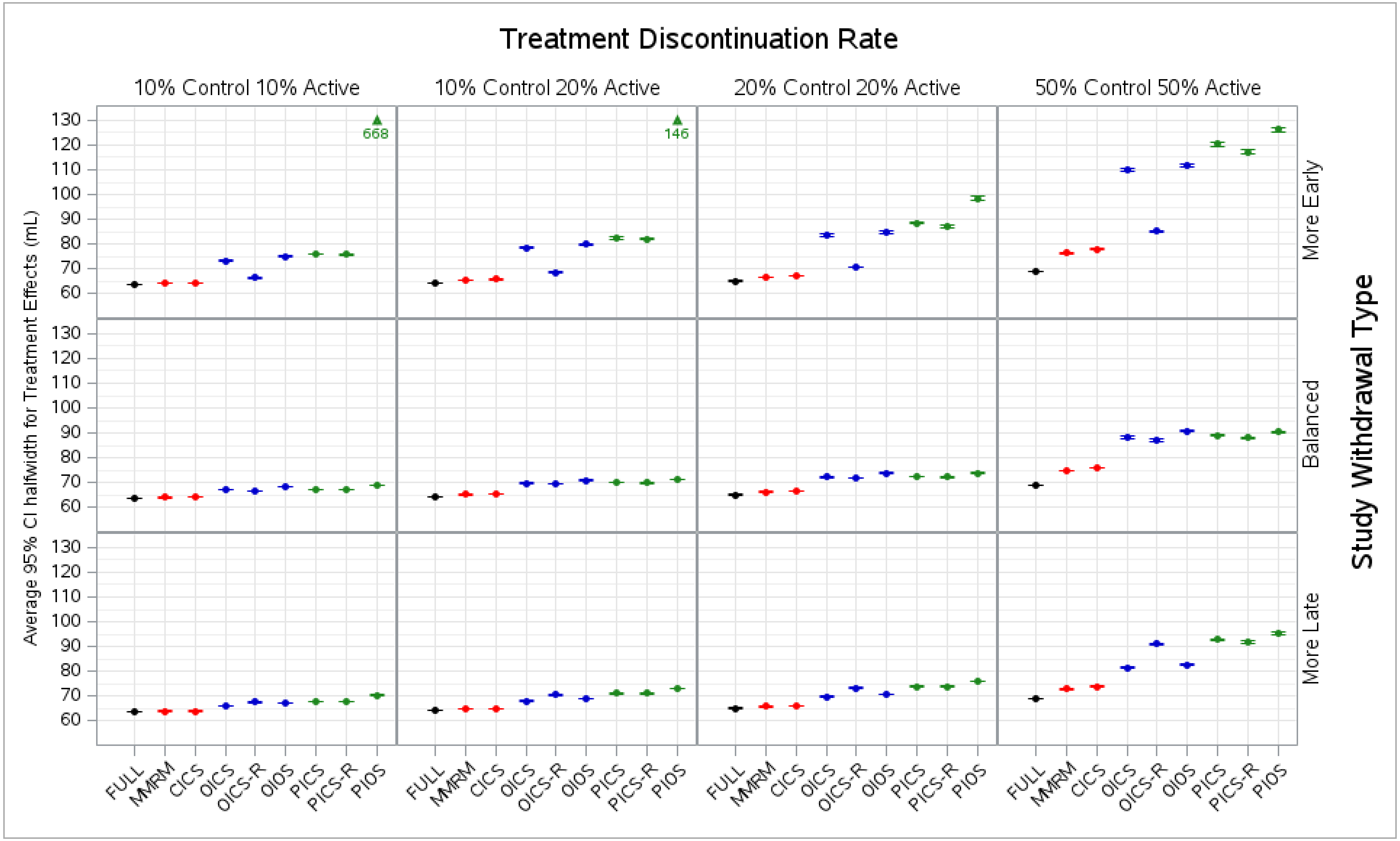

Supplement: Supplementary file 1 [file PST-23-1144-s003.pdf]
