# Supplementary material for: Estimation of Treatment Policy Estimands for Continuous Outcomes Using Off‐Treatment Sequential Multiple Imputation
Source: Pharm Stat. 2024 Aug 4;23(6):1144–55. doi: 10.1002/pst.2411 (PMC11602932; doi:10.1002/pst.2411)

SI Figure 3.1
95% CI Coverage for Treatment Estimates (Active vs. Control)

Disc. Scenario: Return To Baseline
Disc. Mechanism: DAR
With. Rate: 50%

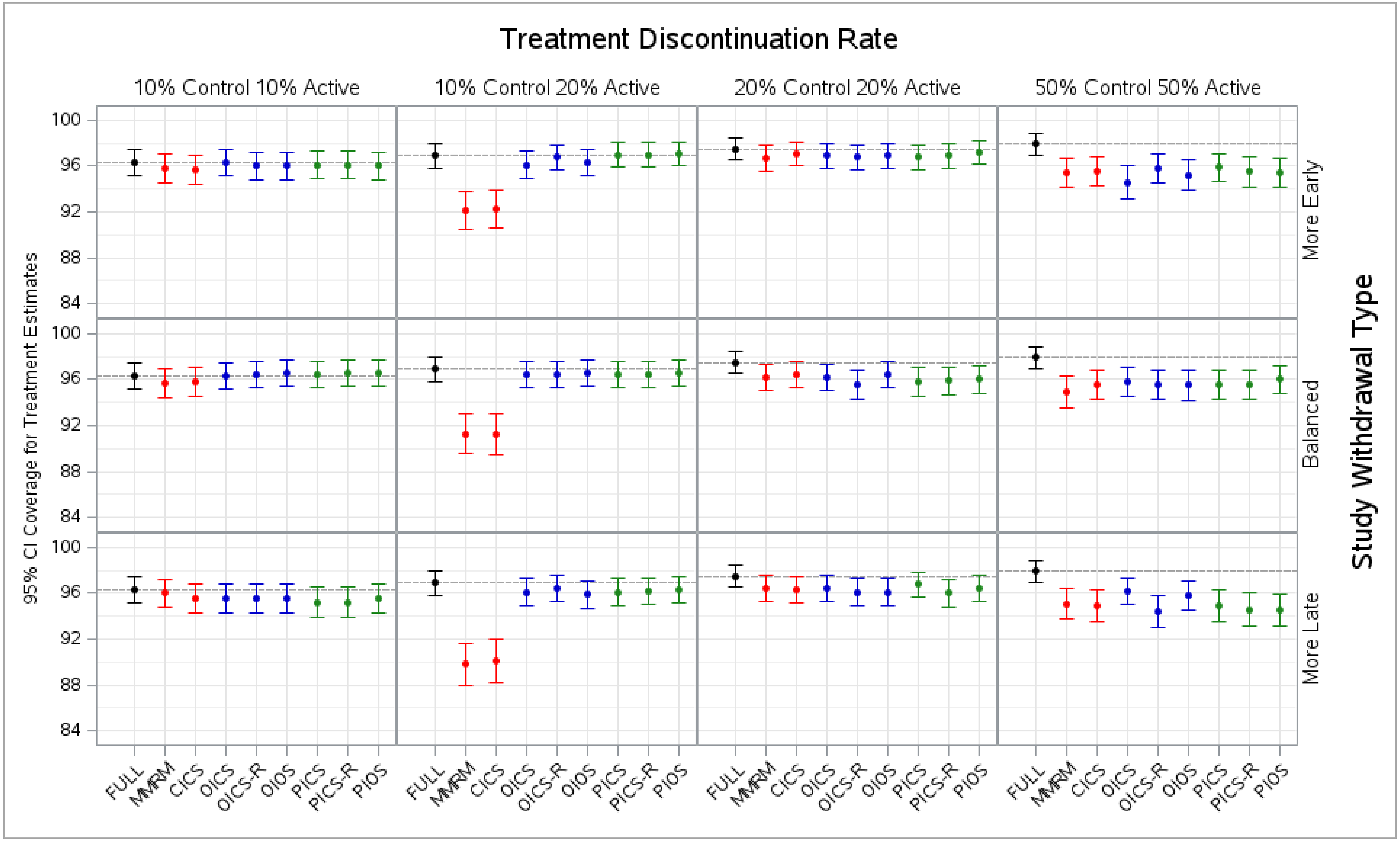

SI Figure 3.2
95% CI Coverage for Treatment Estimates (Active vs. Control)

Disc. Scenario: Return To Baseline
Disc. Mechanism: DAR
With. Rate: 70%

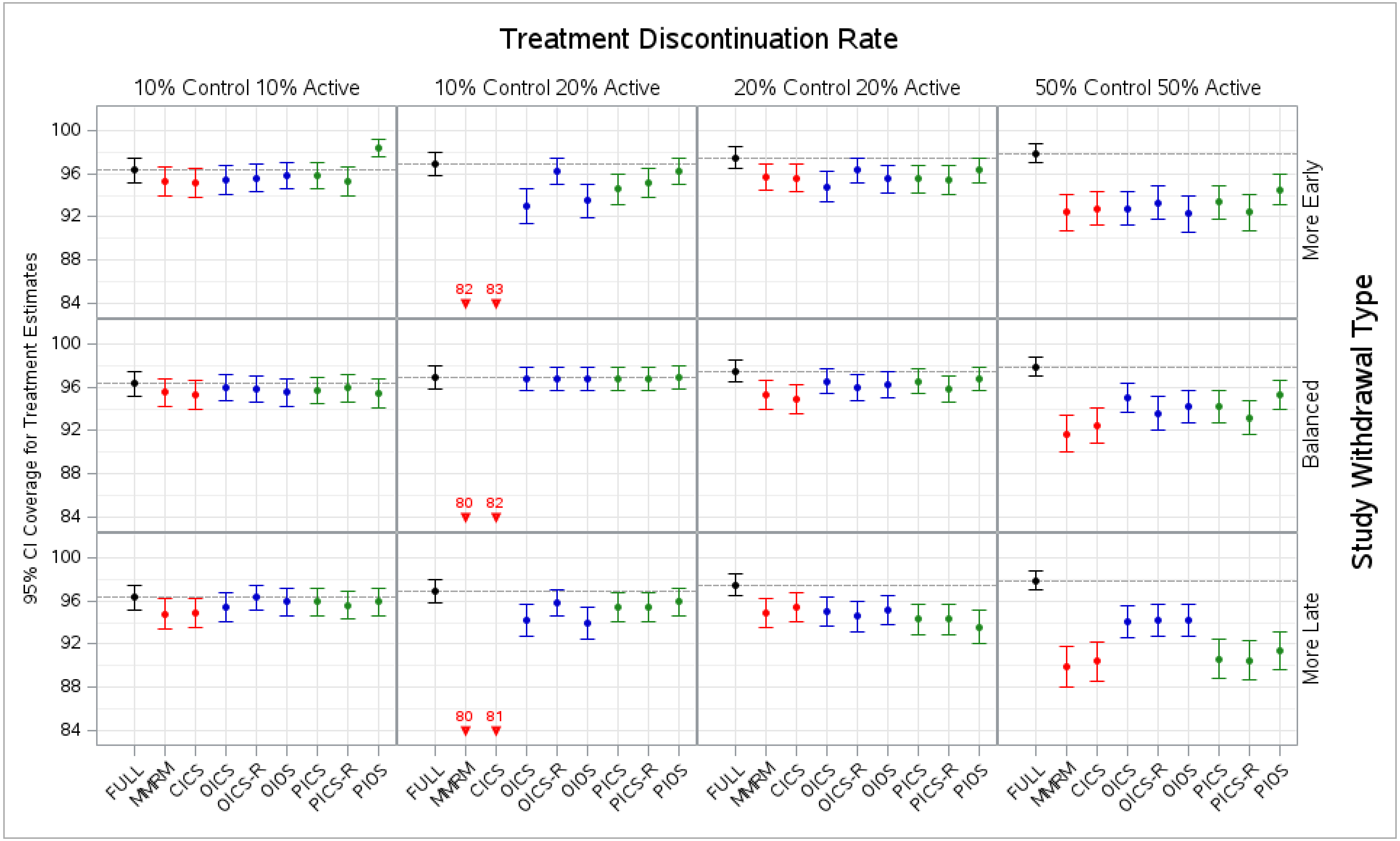

SI Figure 3.3
95% CI Coverage for Treatment Estimates (Active vs. Control)

Disc. Scenario: Same As Active
Disc. Mechanism: DAR
With. Rate: 50%

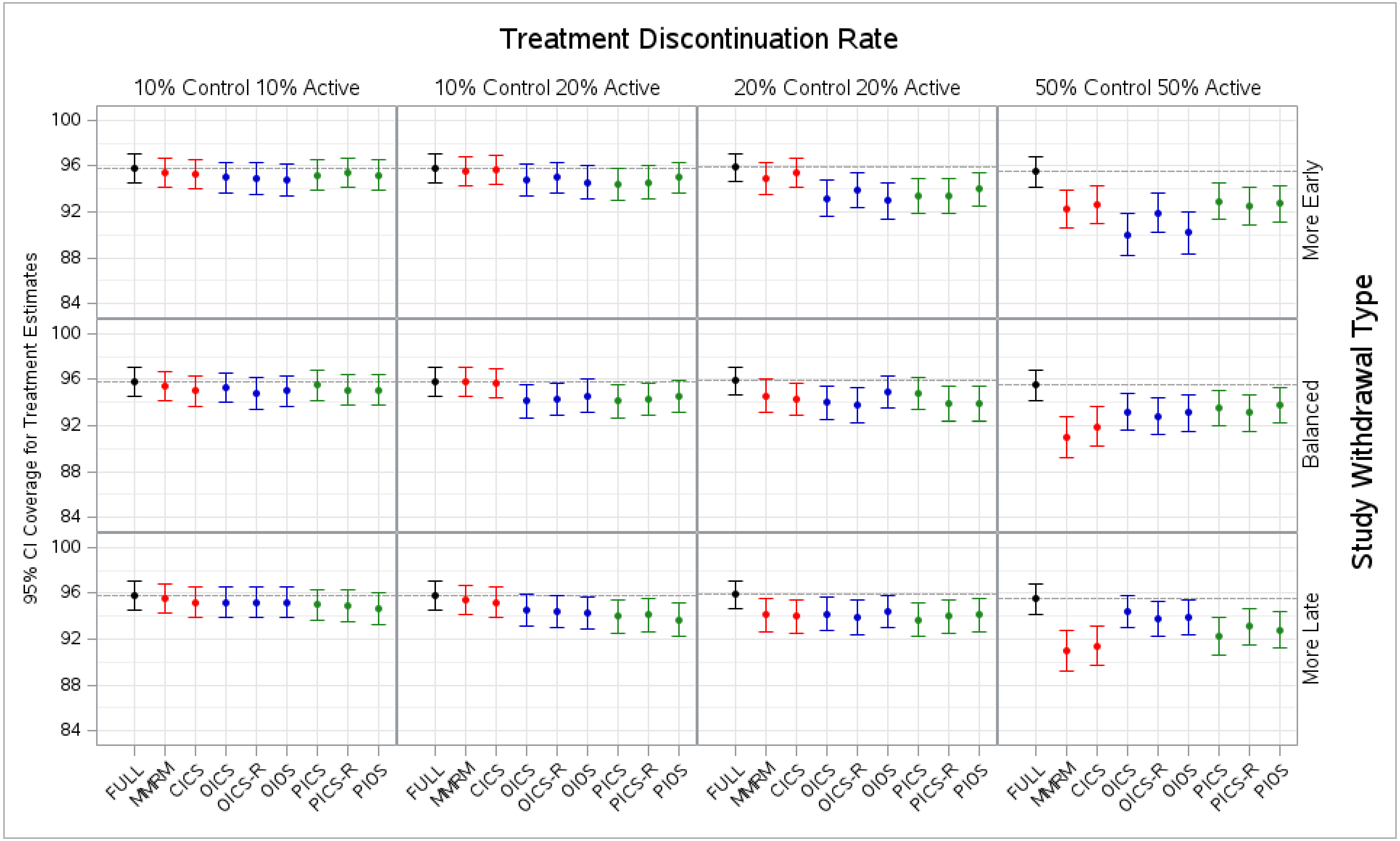

SI Figure 3.4
95% CI Coverage for Treatment Estimates (Active vs. Control)

Disc. Scenario: Same As Active
Disc. Mechanism: DAR
With. Rate: 70%

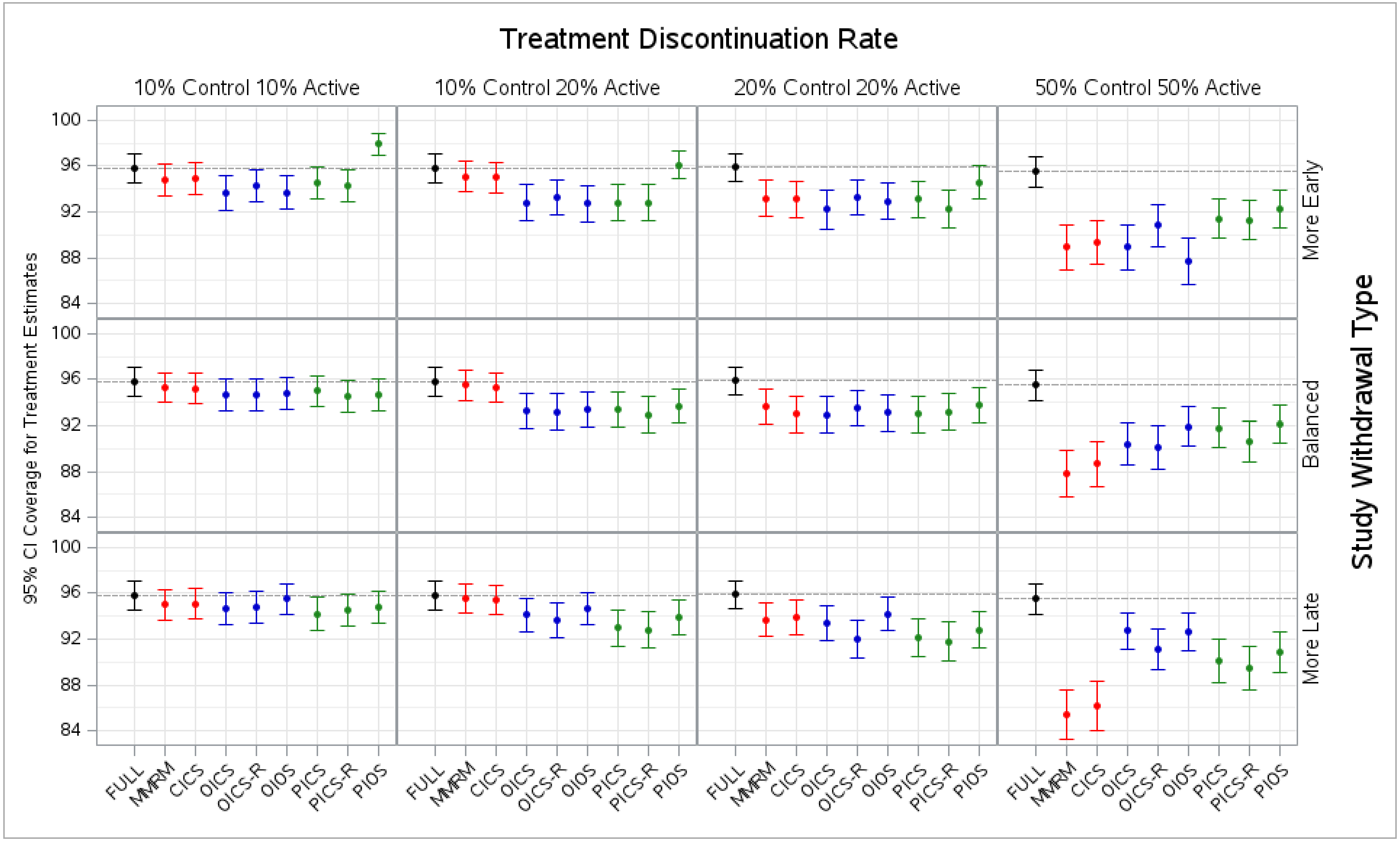

SI Figure 3.5
95% CI Coverage for Treatment Estimates (Active vs. Control)

Disc. Scenario: Return To Baseline
Disc. Mechanism: DNAR
With. Rate: 50%

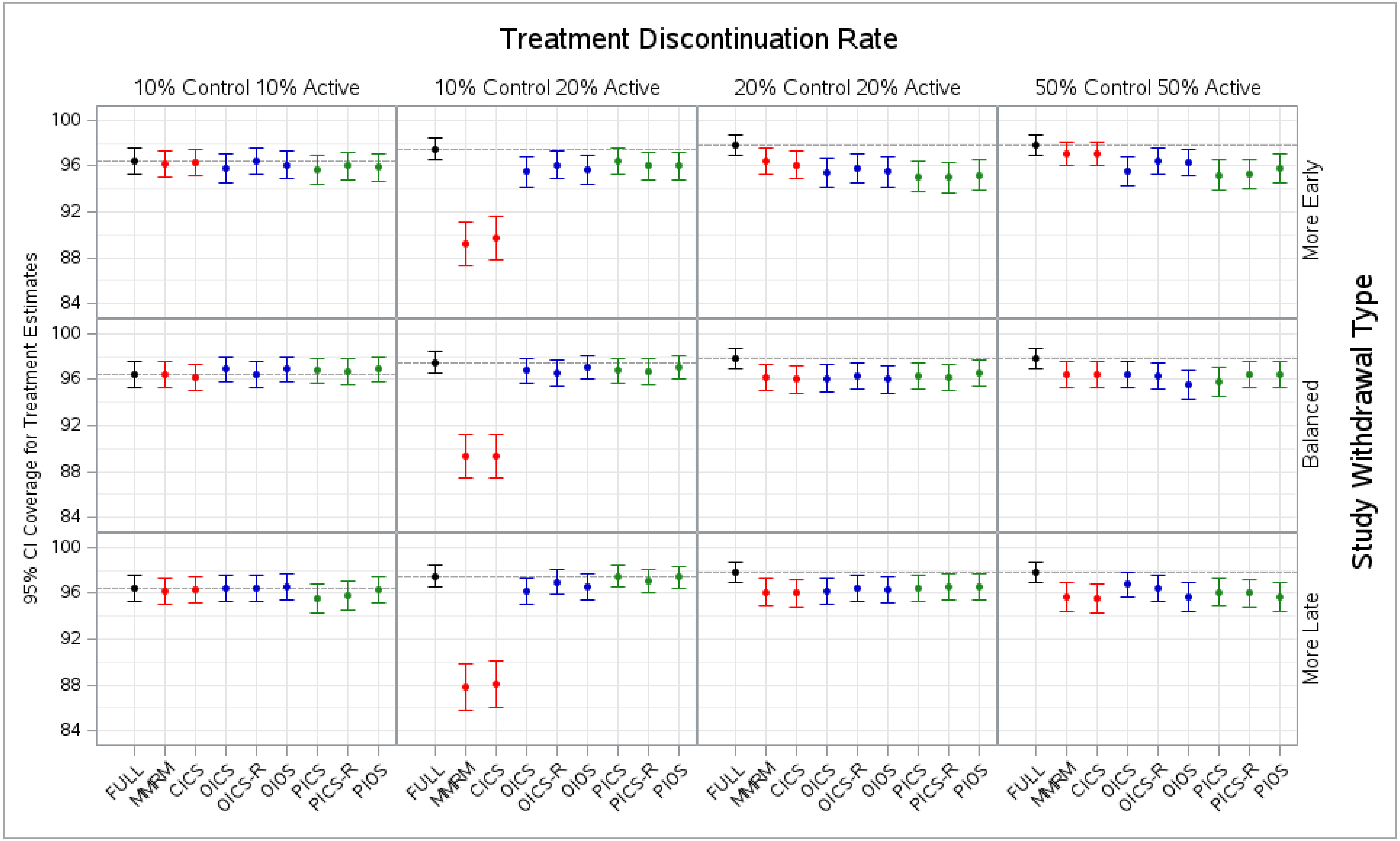

SI Figure 3.6
95% CI Coverage for Treatment Estimates (Active vs. Control)

Disc. Scenario: Return To Baseline
Disc. Mechanism: DNAR
With. Rate: 70%

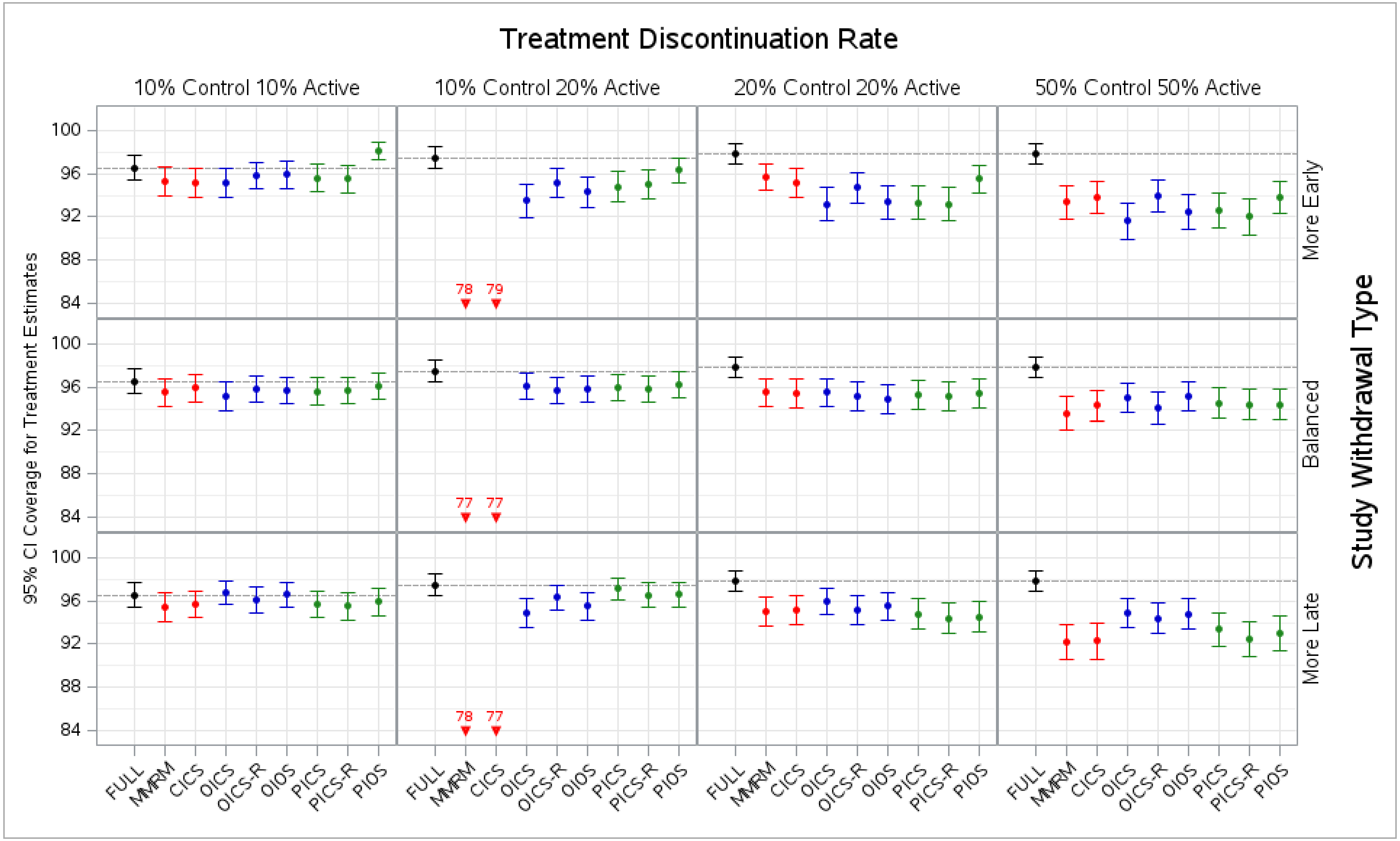

SI Figure 3.7
95% CI Coverage for Treatment Estimates (Active vs. Control)

Disc. Scenario: Same As Active
Disc. Mechanism: DNAR
With. Rate: 50%

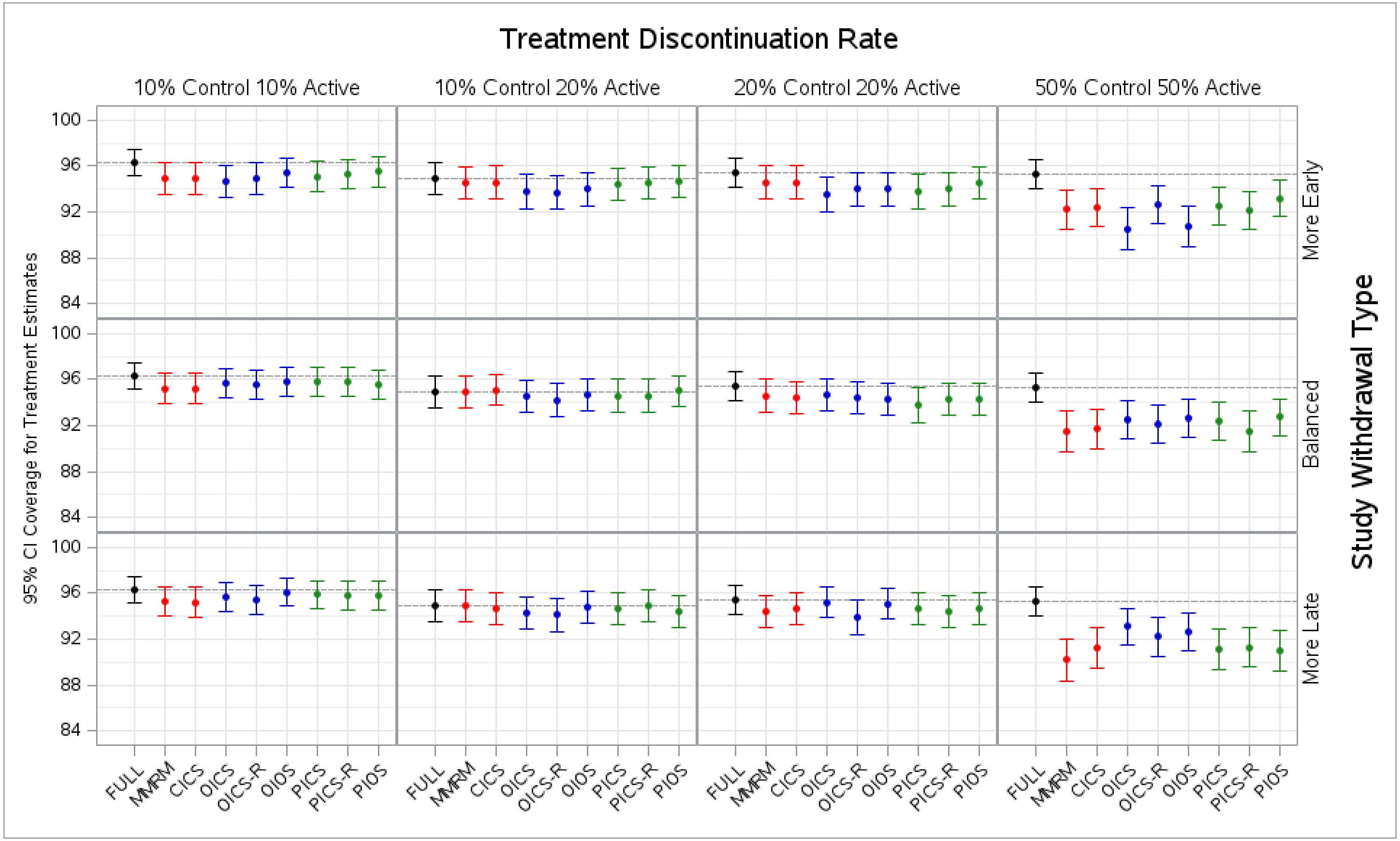

SI Figure 3.8
95% CI Coverage for Treatment Estimates (Active vs. Control)

Disc. Scenario: Same As Active
Disc. Mechanism: DNAR
With. Rate: 70%

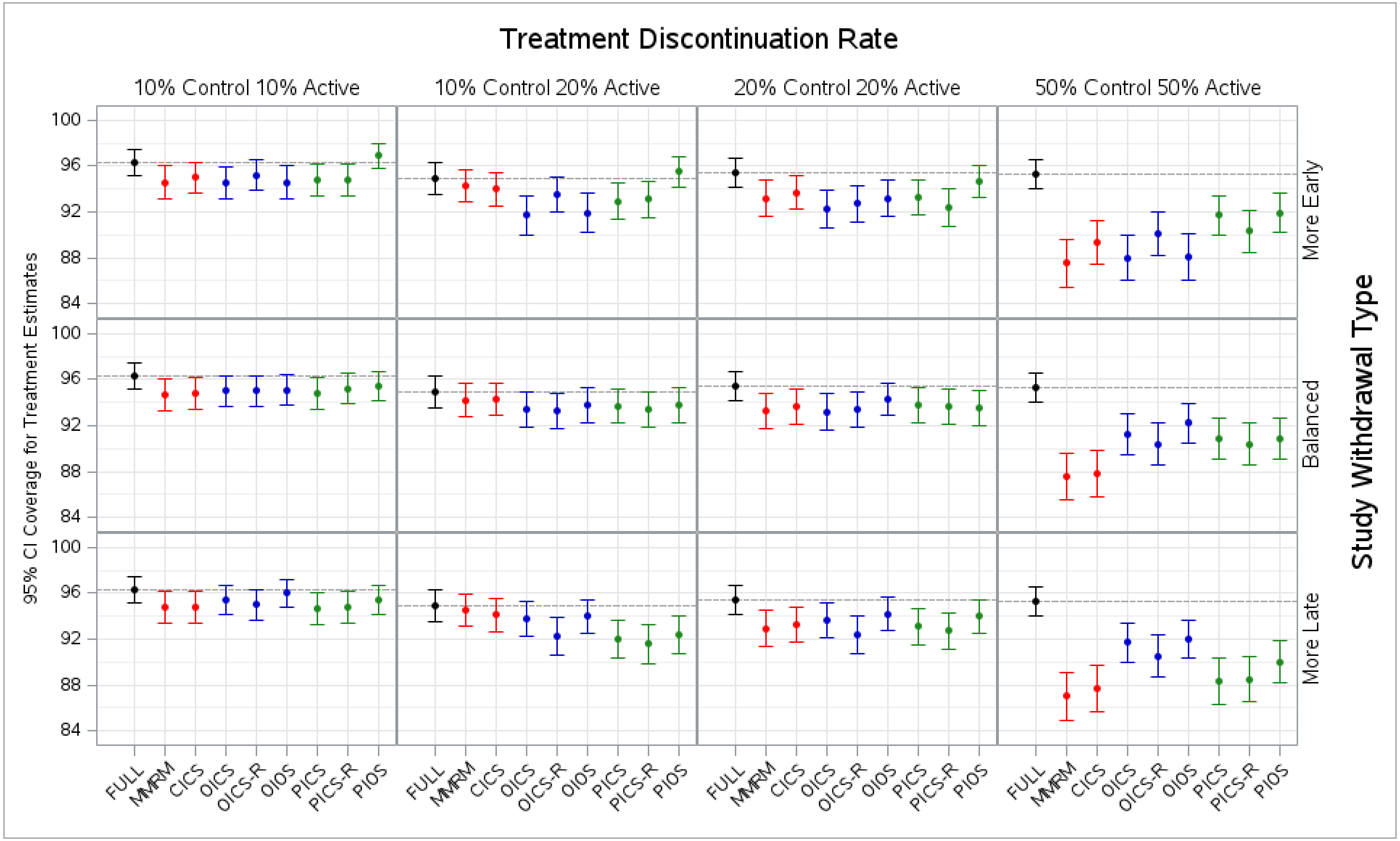

Supplement: Supplementary file 2 [file PST-23-1144-s004.pdf]
