# Supplementary material for: Estimation of Treatment Policy Estimands for Continuous Outcomes Using Off‐Treatment Sequential Multiple Imputation
Source: Pharm Stat. 2024 Aug 4;23(6):1144–55. doi: 10.1002/pst.2411 (PMC11602932; doi:10.1002/pst.2411)
Supplement: Supplementary file 3 [file PST-23-1144-s006.pdf]

SI4 – Type 1 Error (%)

4.1 Return to Baseline – DAR.

Discontinuation Rate	Withdrawal Rate	Withdrawal Type	N	FULL	MMRM	CICS	OICS	OICS-R	OIOS	PICS	PICS-R	PIOS
10% Control 10% Active	50%	More Early	1000	3.3	3.6	3.7	4.6	4.1	3.9	4.0	4.4	3.5
		Balanced	1000	3.3	3.5	3.6	4.1	4.1	4.1	4.1	3.9	3.9
		More Late	1000	3.3	3.7	3.5	3.8	3.9	3.8	4.2	4.0	3.7
	70%	More Early	1000	3.3	4.0	4.0	5.9	4.4	5.3	5.0	5.4	
		Balanced	1000	3.3	3.9	3.8	4.8	4.7	4.4	4.4	4.7	4.5
		More Late	1000	3.3	4.2	4.3	4.5	4.5	3.9	4.9	5.0	4.3
20% Control 20% Active	50%	More Early	1000	3.7	4.4	4.0	4.0	4.9	4.2	4.8	4.5	4.6
		Balanced	1000	3.7	4.4	4.4	4.6	4.9	5.0	4.6	5.0	4.3
		More Late	1000	3.7	4.3	4.4	4.6	4.4	4.4	4.9	4.4	4.6
	70%	More Early	1000	3.7	4.6	4.5	7.0	6.0	6.7	7.0	6.5	5.1
		Balanced	1000	3.7	5.2	5.1	5.5	5.8	5.3	5.4	5.4	4.9
		More Late	1000	3.7	4.9	4.7	5.6	5.8	5.2	6.8	6.5	6.8
50% Control 50% Active	50%	More Early	1000	2.7	2.4	2.8	4.7	4.8	4.6	4.0	5.0	4.6
		Balanced	1000	2.7	3.1	3.0	3.4	3.6	3.5	3.6	3.5	3.9
		More Late	1000	2.7	3.0	3.1	3.9	3.7	4.3	5.1	5.2	5.3
	70%	More Early	1000	2.7	2.9	2.8	7.0	5.3	6.6	6.7	8.4	6.4
		Balanced	1000	2.7	2.5	2.7	5.9	6.2	5.9	5.9	6.3	5.3
		More Late	1000	2.7	3.7	4.1	5.0	7.1	5.5	8.0	8.5	8.3

4.2: Same as Active – DAR.

Discontinuation Rate	Withdrawal Rate	Withdrawal Type	N	FULL	MMRM	CICS	OICS	OICS-R	OIOS	PICS	PICS-R	PIOS
10% Control 10% Active	50%	More Early	1000	4.7	5.1	4.8	5.8	5.4	6.1	5.7	5.7	5.4
		Balanced	1000	4.7	4.9	4.7	5.2	5.4	5.1	5.3	5.3	5.0
		More Late	1000	4.7	5.3	5.3	5.3	5.1	4.7	5.2	5.1	4.8
	70%	More Early	1000	4.7	4.5	4.6	7.0	6.9	6.5	5.8	5.9	
		Balanced	1000	4.7	4.8	4.7	5.9	6.4	5.3	6.1	6.6	5.9
		More Late	1000	4.7	4.5	5.0	4.2	4.8	3.8	5.6	5.7	4.9
20% Control 20% Active	50%	More Early	1000	4.8	4.0	4.0	5.8	4.8	5.3	5.7	6.3	5.3
		Balanced	1000	4.8	4.6	4.7	5.8	6.3	5.9	6.0	5.8	6.0
		More Late	1000	4.8	4.0	4.0	5.1	6.0	5.7	6.5	5.8	6.2
	70%	More Early	1000	4.8	4.5	4.5	6.6	5.7	6.7	6.8	6.6	5.4
		Balanced	1000	4.8	4.9	4.9	6.2	5.8	5.5	5.9	5.8	5.9
		More Late	1000	4.8	4.6	4.6	5.9	6.9	5.3	8.2	7.3	6.8
50% Control 50% Active	50%	More Early	1000	4.5	4.0	3.9	6.0	5.7	6.1	6.0	6.5	5.8
		Balanced	1000	4.5	4.0	3.7	5.1	5.8	5.7	5.9	5.6	5.4
		More Late	1000	4.5	4.5	4.0	6.0	7.3	5.4	6.9	8.0	6.8
	70%	More Early	1000	4.5	4.6	3.9	7.8	8.1	7.8	7.0	7.7	5.3
		Balanced	1000	4.5	4.4	3.9	8.3	8.4	7.2	7.4	8.4	7.3
		More Late	1000	4.5	4.1	3.8	6.5	9.1	6.0	10.3	11.4	11.3

4.3: Return to Baseline – DNAR.

Discontinuation Rate	Withdrawal Rate	Withdrawal Type	N	FULL	MMRM	CICS	OICS	OICS-R	OIOS	PICS	PICS-R	PIOS
10% Control 10% Active	50%	More Early	1000	3.8	4.1	3.9	3.8	3.7	4.0	4.2	3.7	3.9
		Balanced	1000	3.8	4.5	4.5	4.5	4.8	4.2	4.4	4.3	4.2
		More Late	1000	3.8	4.5	4.5	4.2	4.4	4.4	4.6	4.2	4.6
	70%	More Early	1000	3.8	3.8	3.9	3.7	3.9	4.4	3.9	3.8	
		Balanced	1000	3.8	4.4	4.3	3.4	3.4	3.3	3.3	3.3	3.9
		More Late	1000	3.8	4.7	4.4	5.2	5.6	5.0	5.2	5.8	5.6
20% Control 20% Active	50%	More Early	1000	2.8	3.2	3.3	3.5	3.8	3.7	4.3	4.9	4.0
		Balanced	1000	2.8	3.2	3.4	3.7	3.7	3.7	3.5	3.5	3.5
		More Late	1000	2.8	3.9	3.6	4.2	3.9	3.9	3.7	4.3	3.5
	70%	More Early	1000	2.8	4.8	5.3	4.6	5.3	5.0	4.6	5.0	4.1
		Balanced	1000	2.8	4.1	4.1	4.2	4.8	4.7	4.3	4.9	4.7
		More Late	1000	2.8	4.2	4.2	3.9	5.0	4.4	7.0	6.5	5.5
50% Control 50% Active	50%	More Early	1000	2.8	2.4	2.5	4.7	4.1	4.2	4.6	4.6	4.6
		Balanced	1000	2.8	3.2	3.1	4.7	5.6	4.3	5.0	5.4	4.8
		More Late	1000	2.8	3.5	3.7	4.2	4.5	4.1	5.2	5.1	5.6
	70%	More Early	1000	2.8	2.6	2.9	6.7	6.8	7.0	6.3	7.3	6.2
		Balanced	1000	2.8	3.0	3.2	6.0	6.1	5.8	6.4	6.5	5.9
		More Late	1000	2.8	3.5	3.4	5.3	5.3	4.7	7.4	7.5	7.9

4.4: Same as Active – DNAR.

Discontinuation Rate	Withdrawal Rate	Withdrawal Type	N	FULL	MMRM	CICS	OICS	OICS-R	OIOS	PICS	PICS-R	PIOS
10% Control 10% Active	50%	More Early	1000	4.5	3.7	3.7	4.4	4.6	4.1	4.9	4.8	4.7
		Balanced	1000	4.5	3.7	3.8	4.4	4.3	4.0	4.1	4.5	4.1
		More Late	1000	4.5	4.2	4.2	4.2	4.3	4.5	4.3	4.1	4.2
	70%	More Early	1000	4.5	4.5	4.4	5.9	4.8	5.2	5.7	5.4	
		Balanced	1000	4.5	4.2	4.0	4.8	4.7	4.7	5.0	4.7	4.1
		More Late	1000	4.5	3.9	4.1	4.7	5.0	4.6	5.0	5.1	4.2
20% Control 20% Active	50%	More Early	1000	3.8	5.1	5.4	7.4	7.0	7.5	7.5	7.2	6.8
		Balanced	1000	3.8	4.4	4.2	6.0	5.9	5.3	6.1	5.9	5.8
		More Late	1000	3.8	5.0	5.0	4.6	5.6	4.9	5.7	6.0	5.8
	70%	More Early	1000	3.8	4.7	4.7	7.6	6.3	7.1	6.5	6.9	4.7
		Balanced	1000	3.8	5.8	5.6	8.0	7.3	7.0	7.5	7.8	7.3
		More Late	1000	3.8	4.6	4.7	6.6	7.3	6.0	7.5	7.7	6.8
50% Control 50% Active	50%	More Early	1000	4.9	4.1	4.5	6.5	6.6	7.0	6.6	7.4	6.3
		Balanced	1000	4.9	5.1	5.1	6.4	7.5	6.4	6.8	7.3	6.4
		More Late	1000	4.9	4.6	4.3	5.8	6.9	5.4	7.1	7.7	7.0
	70%	More Early	1000	4.9	4.7	4.3	10.2	8.1	9.8	7.6	7.7	6.7
		Balanced	1000	4.9	4.8	4.8	7.6	8.5	7.8	8.1	8.4	6.8
		More Late	1000	4.9	5.0	4.4	6.6	8.9	6.9	11.4	11.6	11.1
