# Supplementary material for: Estimation of Treatment Policy Estimands for Continuous Outcomes Using Off‐Treatment Sequential Multiple Imputation
Source: Pharm Stat. 2024 Aug 4;23(6):1144–55. doi: 10.1002/pst.2411 (PMC11602932; doi:10.1002/pst.2411)
Supplement: Supplementary file 4 [file PST-23-1144-s007.pdf]

SI Figure 1.1
Bias for Treatment Effects - (Active vs. Control)

Disc. Scenario: Return To Baseline
Disc. Mechanism: DAR
With. Rate: 50%

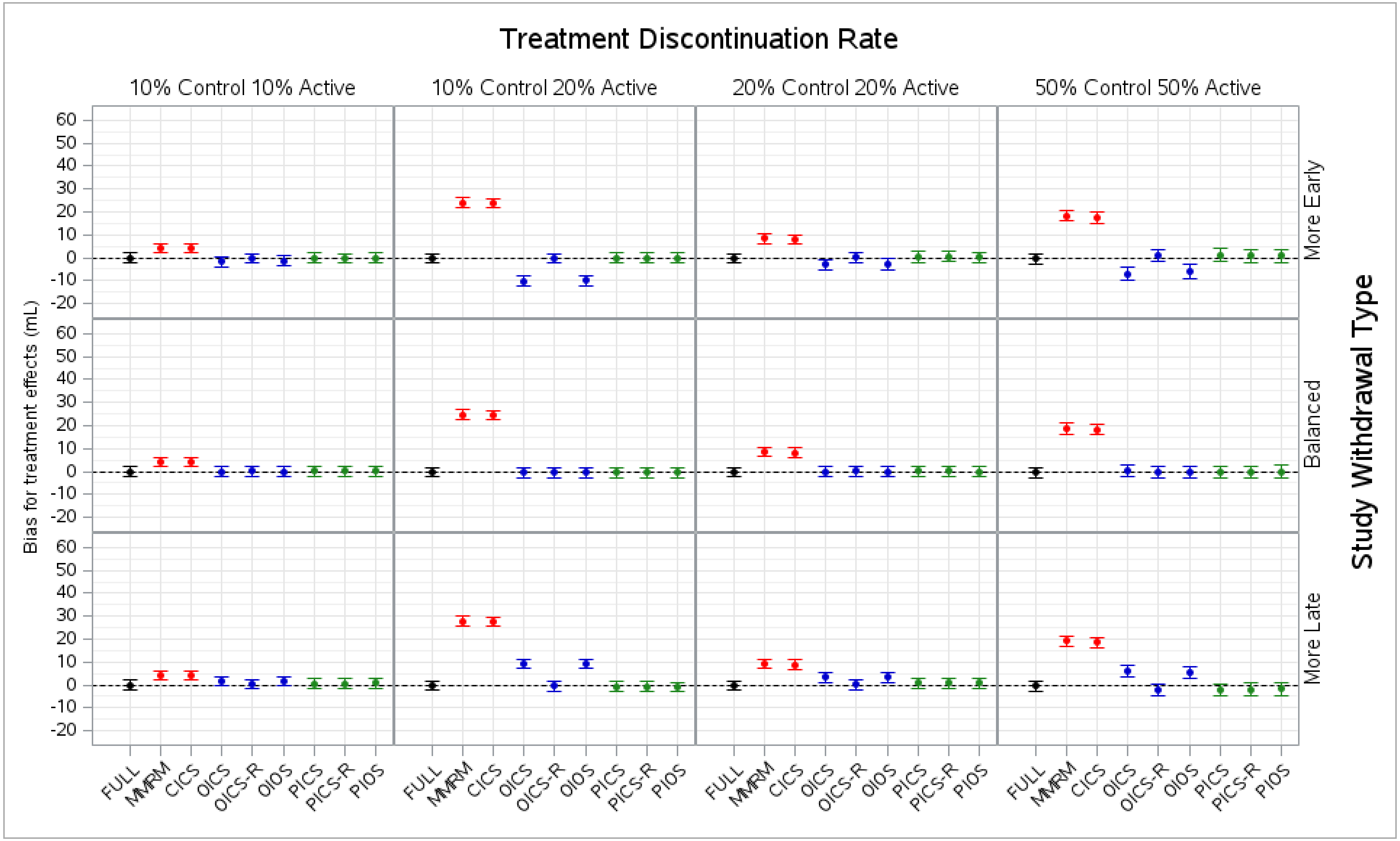

SI Figure 1.2
Bias for Treatment Effects - (Active vs. Control)

Disc. Scenario: Return To Baseline
Disc. Mechanism: DAR
With. Rate: 70%

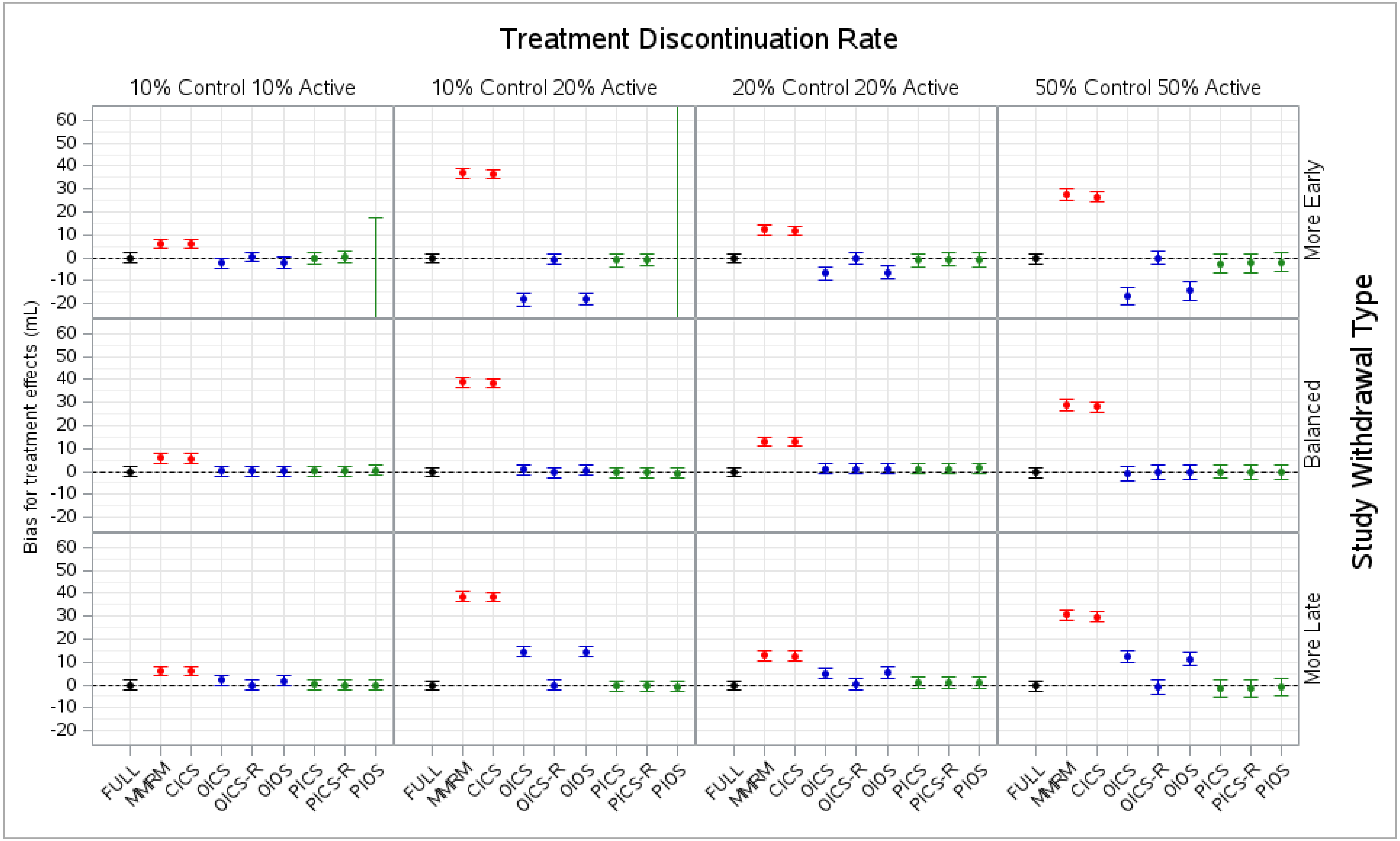

Note: PIOS Bias (95% CI) values: 10% Control 10% Active, More Early: -31.5 (-82.6, 19.6) and 10% Control 20% Active, More Early: -186.2 (-561.6, 189.2).

SI Figure 1.3
Bias for Treatment Effects - (Active vs. Control)

Disc. Scenario: Same As Active
Disc. Mechanism: DAR
With. Rate: 50%

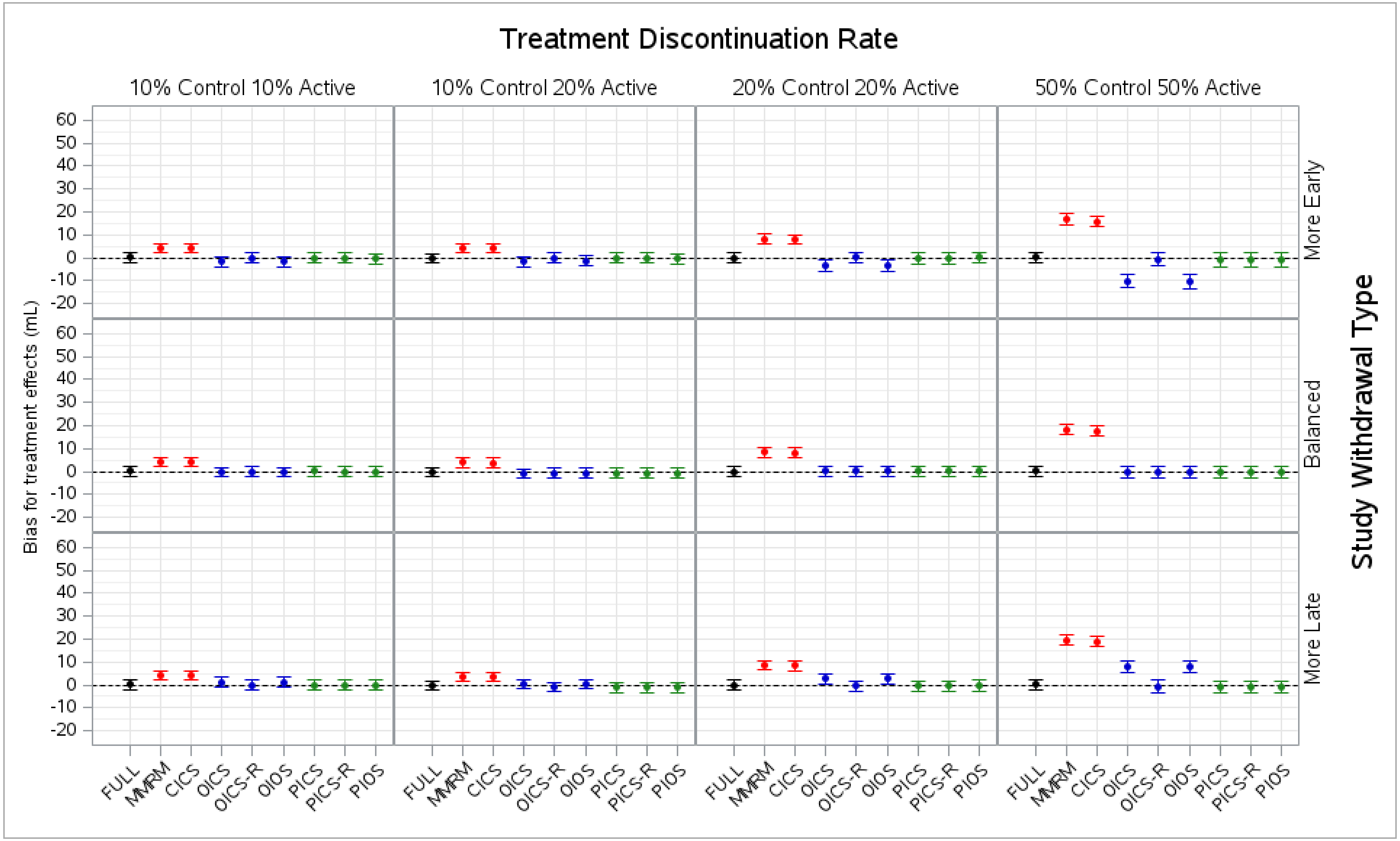

SI Figure 1.4
Bias for Treatment Effects - (Active vs. Control)

Disc. Scenario: Same As Active
Disc. Mechanism: DAR
With. Rate: 70%

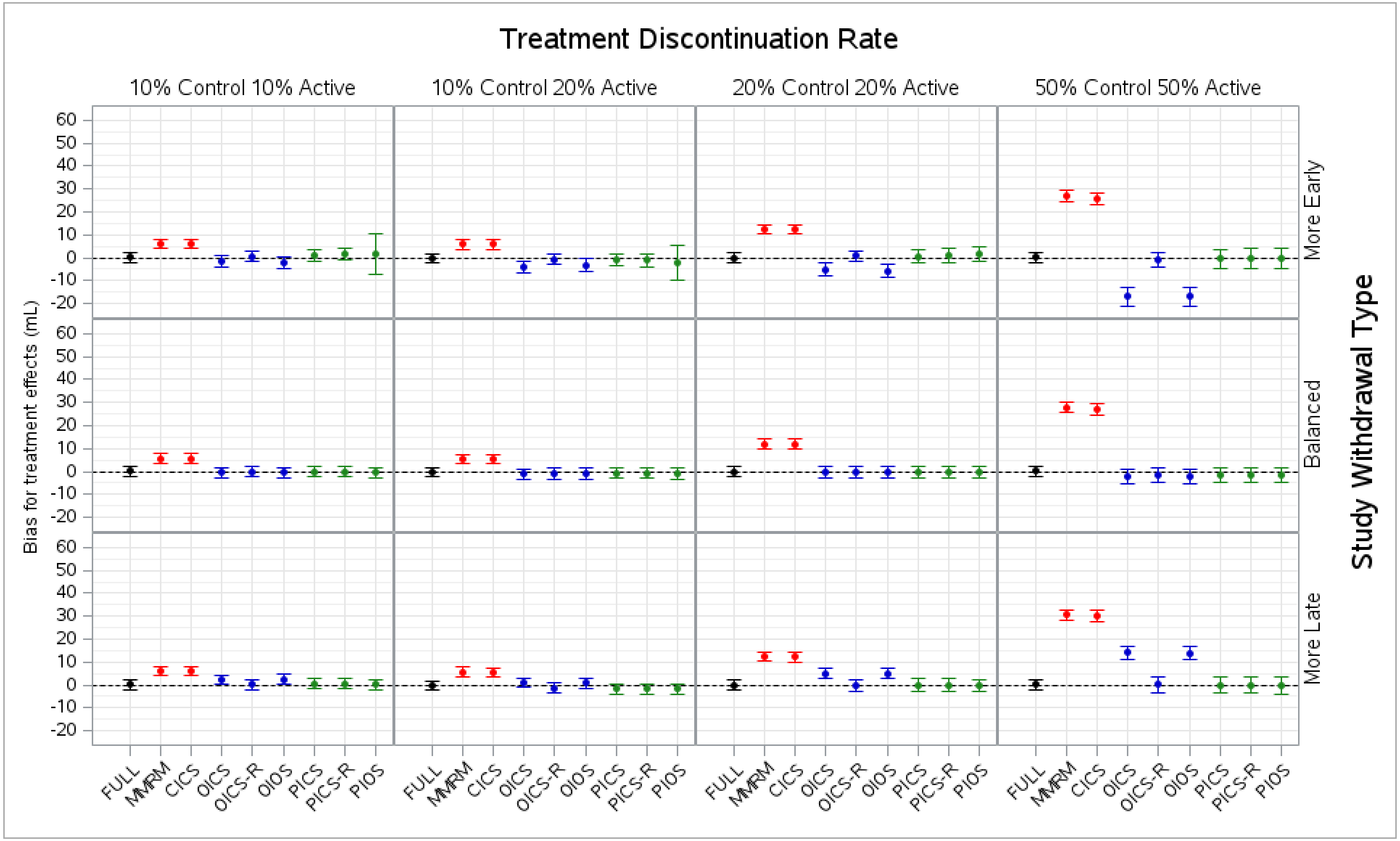

SI Figure 1.5
Bias for Treatment Effects - (Active vs. Control)

Disc. Scenario: Return To Baseline
Disc. Mechanism: DNAR
With. Rate: 50%

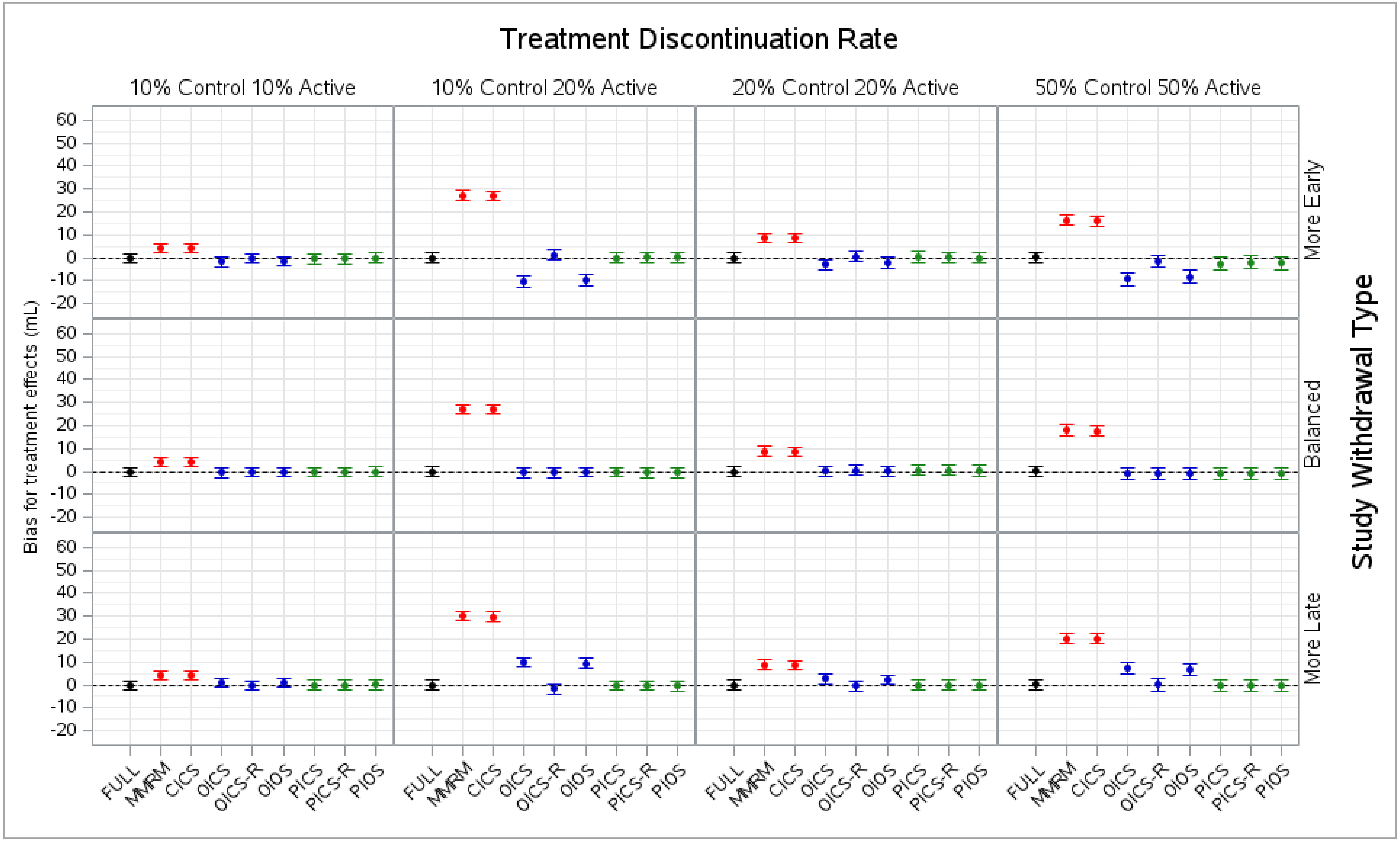

SI Figure 1.6
Bias for Treatment Effects - (Active vs. Control)

Disc. Scenario: Return To Baseline
Disc. Mechanism: DNAR
With. Rate: 70%

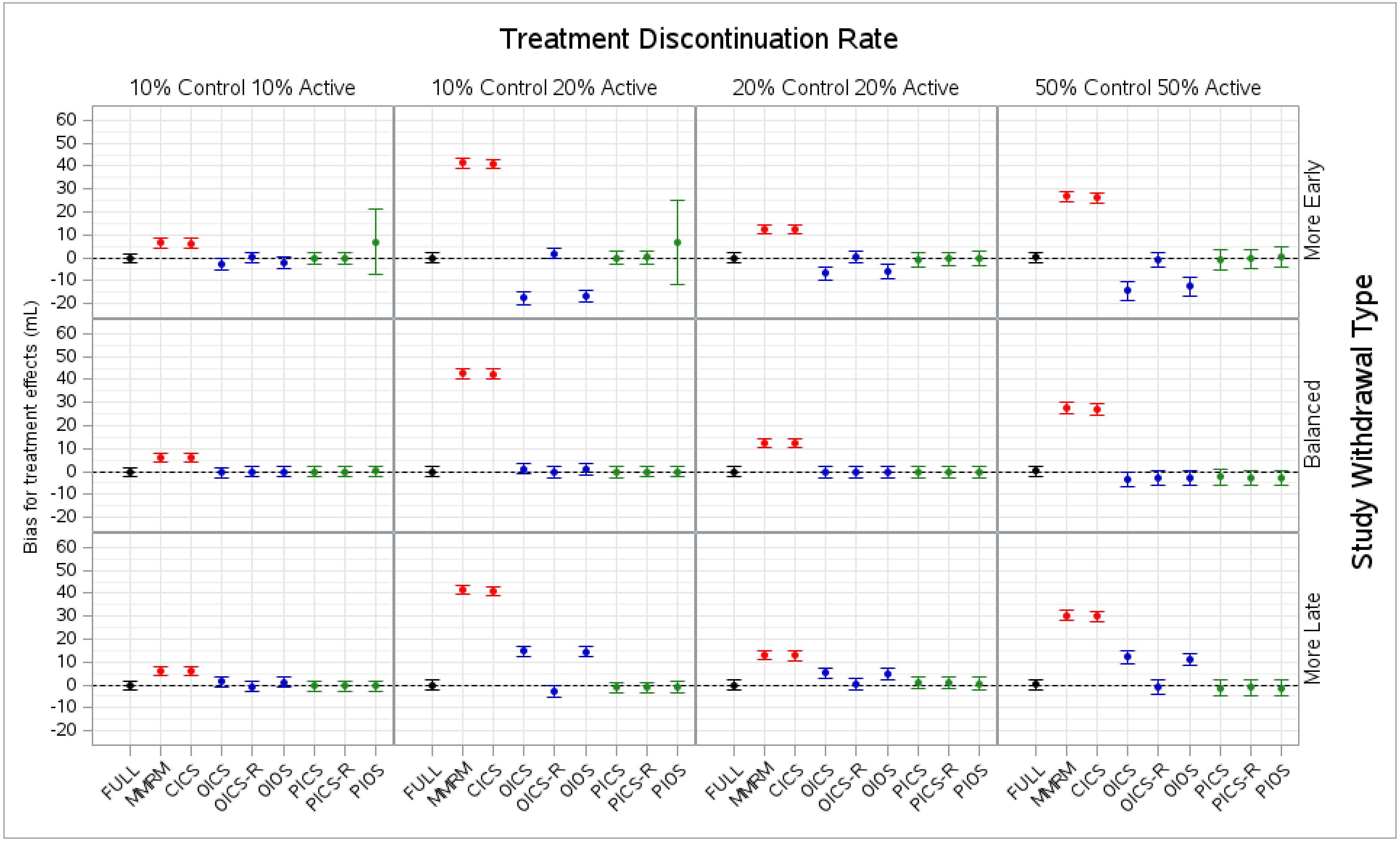

SI Figure 1.7
Bias for Treatment Effects - (Active vs. Control)

Disc. Scenario: Same As Active
Disc. Mechanism: DNAR
With. Rate: 50%

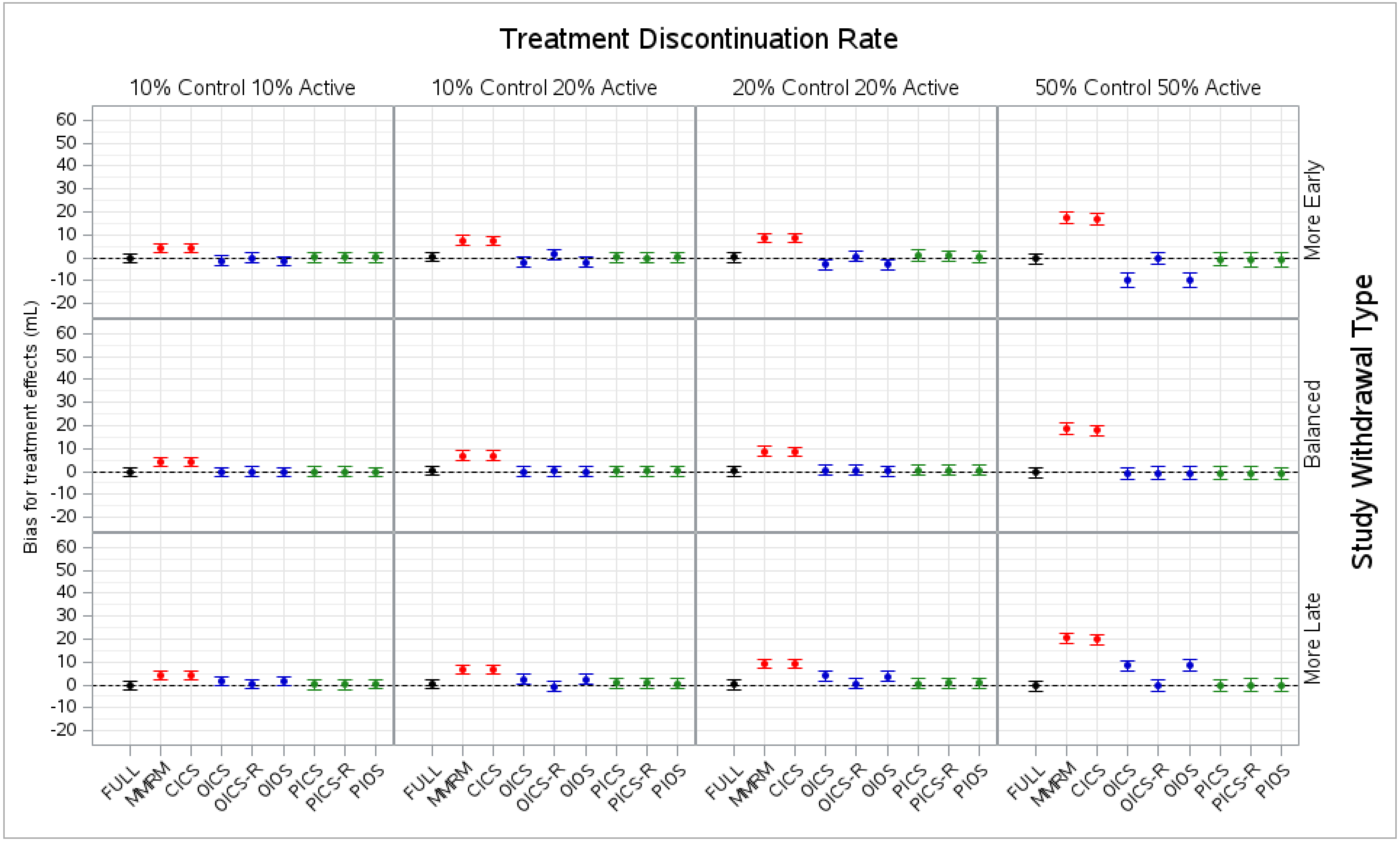

SI Figure 1.8
Bias for Treatment Effects - (Active vs. Control)

Disc. Scenario: Same As Active
Disc. Mechanism: DNAR
With. Rate: 70%

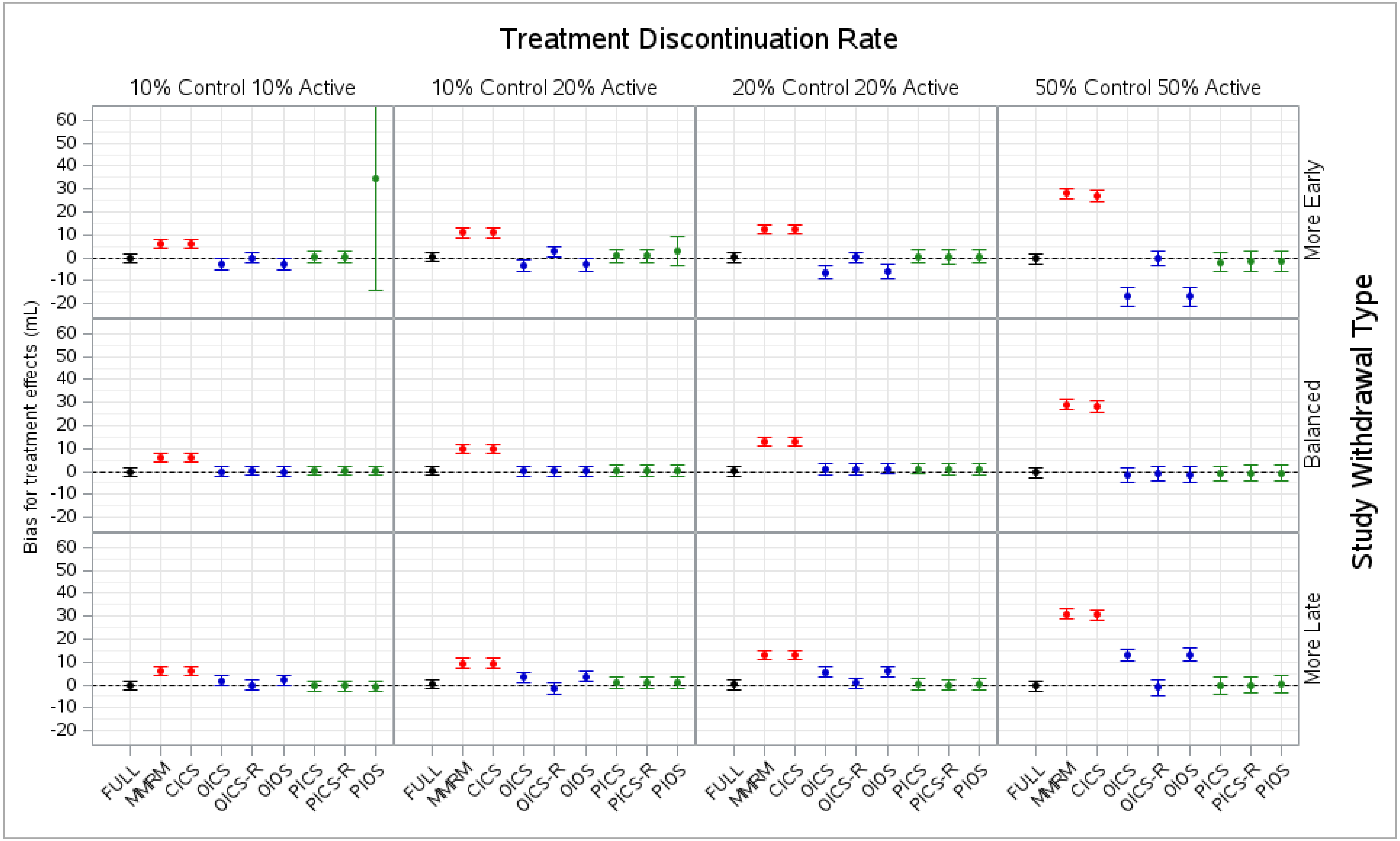

Note: PIOS Bias (95% CI) values: 10% Control 10% Active, More Early: 34.2 (-14.6, 83.0).
