# Supplementary material for: Estimation of Treatment Policy Estimands for Continuous Outcomes Using Off‐Treatment Sequential Multiple Imputation
Source: Pharm Stat. 2024 Aug 4;23(6):1144–55. doi: 10.1002/pst.2411 (PMC11602932; doi:10.1002/pst.2411)
Supplement: Supplementary file 5 [file PST-23-1144-s001.docx]

Appendix 1: MI Models

# 1 MI model descriptions

# 2 Comparison of OICS and OICS-R

The following notes illustrate that OICS and OICS-R may appear to be the same but are in fact different imputation models. We show this by comparing the expected values for imputing the different discontinuation patterns. The OICS and OICS-R models are defined as:

| **OICS** | **OICS-R** |
| --- | --- |
| $Y_{1}=α_{10}+D_{1}∙α_{11}+β_{10}∙Y_{0}$ | $Y_{1}=μ_{1}+D_{1}∙δ_{1}+β_{10}∙R_{0}$ |
| $Y_{2}=α_{20}+D_{2}∙α_{21}+β_{20}∙Y_{0}+β_{21}∙Y_{1}$ | $Y_{2}=μ_{2}+D_{2}∙δ_{2}+β_{20}∙R_{0}+β_{21}∙R_{1}$ |
| $Y_{3}=α_{30}+D_{3}∙α_{31}+β_{30}∙Y_{0}+β_{31}∙Y_{1}+β_{32}∙Y_{2}$ | $Y_{3}=μ_{3}+D_{3}∙δ_{3}+β_{30}∙R_{0}+β_{31}∙R_{1}+β_{32}∙R_{2}$ |

Letting $μ_{0}$ be the baseline expected value and $μ_{j}^{P}$ be the expected value for time point $j$ and pattern $P$ which we specify as combinations of “O” and “X” to represent on- and off-treatment at prior timepoints, the OICS and OICS-R expected values for each pattern are:

| **Mean** | **OICS** | **OICS-R** |
| --- | --- | --- |
| $μ_{1}^{O}$ | $α_{10}+β_{10}∙μ_{0}$ | $μ_{1}$ |
| $μ_{1}^{X}$ | $α_{10}+α_{11}+β_{10}∙μ_{0}$ | $μ_{1}+δ_{1}$ |
| $μ_{2}^{OO}$ | $α_{20}+β_{20}∙μ_{0}+β_{21}(α_{10}+β_{10}∙μ_{0})$ | $μ_{2}$ |
| $μ_{2}^{OX}$ | $α_{20}+α_{21}+β_{20}∙μ_{0}+β_{21}(α_{10}+β_{10}∙μ_{0})$ | $μ_{2}+δ_{2}$ |
| $μ_{2}^{XX}$ | $α_{20}+α_{21}+β_{20}∙μ_{0}+β_{21}(α_{10}+α_{11}+β_{10}∙μ_{0})$ | $μ_{2}+δ_{2}$ |
| $μ_{3}^{OOO}$ | $α_{30}+β_{30}∙μ_{0}+β_{31}(α_{10}+β_{10}∙μ_{0})+β_{32}(α_{20}+β_{20}∙μ_{0}+β_{21}(α_{10}+β_{10}∙μ_{0}))$ | $μ_{3}$ |
| $μ_{3}^{OOX}$ | $α_{30}+α_{31}+β_{30}∙μ_{0}+β_{31}(α_{10}+β_{10}∙μ_{0})+β_{32}(α_{20}+β_{20}∙μ_{0}+β_{21}(α_{10}+β_{10}∙μ_{0}))$ | $μ_{3}+δ_{3}$ |
| $μ_{3}^{OXX}$ | $α_{30}+α_{31}+β_{30}∙μ_{0}+β_{31}(α_{10}+β_{10}∙μ_{0})+β_{32}(α_{20}+α_{21}+β_{20}∙μ_{0}+β_{21}(α_{10}+β_{10}∙μ_{0}))$ | $μ_{3}+δ_{3}$ |
| $μ_{3}^{XXX}$ | $α_{30}+α_{31}+β_{30}∙μ_{0}+β_{31}(α_{10}+α_{11}+β_{10}∙μ_{0})+β_{32}(α_{20}+α_{21}+β_{20}∙μ_{0}+β_{21}(α_{10}+α_{11}+β_{10}∙μ_{0}))$ | $μ_{3}+δ_{3}$ |

The structure of the expected values for OICS-R simplify due to $E(R_{j})=0$ and so we can see the expected values for imputation in OICS depend upon the pattern of discontinuation, whereas the OICS-R model uses the same expected value for imputation across patterns and therefore OICS and OICS-R are not the same.

# 3 Comparison of PICS and PICS-R

The following notes illustrate that the PICS and PICS-R models are essentially the same model but parameterised differently. The PICS and PICS-R models are defined as:

| **PICS** | **PICS-R** |
| --- | --- |
| $Y_{1}=α_{10}+P_{11}∙α_{11}+β_{10}∙Y_{0}$ | $Y_{1}=μ_{1}+P_{11}∙δ_{11}+β_{10}∙R_{0}$ |
| $Y_{2}=α_{20}+P_{21}∙α_{21}+P_{22}∙α_{22}+β_{20}∙Y_{0}+β_{21}∙Y_{1}$ | $Y_{2}=μ_{2}+P_{21}∙δ_{21}+P_{22}∙δ_{22}+β_{20}∙R_{0}+β_{21}∙R_{1}$ |
| $Y_{3}=α_{30}+P_{31}∙α_{31}+P_{32}∙α_{32}+P_{33}∙α_{33}+β_{30}∙Y_{0}+β_{31}∙Y_{1}+β_{32}∙Y_{2}$ | $Y_{3}=μ_{3}+P_{31}∙δ_{31}+P_{32}∙δ_{32}+P_{33}∙δ_{33}+β_{30}∙R_{0}+β_{31}∙R_{1}+β_{32}∙R_{2}$ |

Letting $μ_{0}$ be the baseline expected value and $μ_{j}^{P}$ be the pattern mean for time point $j$ and pattern $P$ which we specify as combinations of “O” and “X” to represent on- and off-treatment at prior timepoints, the PICS and PICS-R expected values for each pattern are:

| **Mean** | **PICS** | **PICS-R** |
| --- | --- | --- |
| $μ_{1}^{O}$ | $α_{10}+β_{10}∙μ_{0}$ | $μ_{1}$ |
| $μ_{1}^{X}$ | $α_{10}+ α_{11}+β_{10}∙μ_{0}$ | $μ_{1}+δ_{11}$ |
| $μ_{2}^{OO}$ | $α_{20}+ β_{20}∙μ_{0}+β_{21}(α_{10}+β_{10}∙μ_{0})$ | $μ_{2}$ |
| $μ_{2}^{OX}$ | $α_{20}+ α_{21}+ β_{20}∙μ_{0}+β_{21}(α_{10}+β_{10}∙μ_{0})$ | $μ_{2}+δ_{21}$ |
| $μ_{2}^{XX}$ | $α_{20}+ α_{22}+ β_{20}∙μ_{0}+β_{21}(α_{10}+α_{11}+β_{10}∙μ_{0})$ | $μ_{2}+δ_{22}$ |
| $μ_{3}^{OOO}$ | $α_{30}+ β_{30}∙μ_{0}+β_{31}(α_{10}+β_{10}{∙μ}_{0})+β_{32}(α_{20}+β_{20}μ_{0}+β_{21}(α_{10}+β_{10}{∙μ}_{0}))$ | $μ_{3}$ |
| $μ_{3}^{OOX}$ | $α_{30}+ α_{31}+ β_{30}∙μ_{0}+β_{31}(α_{10}+β_{10}{∙μ}_{0})+β_{32}(α_{20}+β_{20}μ_{0}+β_{21}(α_{10}+β_{10}{∙μ}_{0}))$ | $μ_{3}+δ_{31}$ |
| $μ_{3}^{OXX}$ | $α_{30}+ α_{32}+ β_{30}∙μ_{0}+β_{31}(α_{10}+β_{10}{∙μ}_{0})+β_{32}(α_{20}+α_{21}+β_{20}μ_{0}+β_{21}(α_{10}+β_{10}{∙μ}_{0}))$ | $μ_{3}+δ_{32}$ |
| $μ_{3}^{XXX}$ | $α_{30}+ α_{33}+ β_{30}∙μ_{0}+β_{31}(α_{10}+α_{11}+β_{10}{∙μ}_{0})+β_{32}(α_{20}+α_{22}+β_{20}μ_{0}+β_{21}(α_{10}+α_{11}+β_{10}{∙μ}_{0}))$ | $μ_{3}+δ_{33}$ |

Equating the expected values between the models for each discontinuation pattern and working from timepoint one using substitution we can see:

Timepoint 1:

$α_{10}=μ_{1}-β_{10}∙μ_{0}$

$α_{11}=δ_{11}$

Timepoint 2:

$α_{20}=μ_{2}-β_{20}∙μ_{0}-β_{21}∙μ_{1}$

$α_{21}=δ_{21}$

$α_{22}=δ_{22}-β_{21}∙δ_{11}$

Timepoint 3:

$α_{30}=μ_{3}-β_{30}∙μ_{0}-β_{31}∙μ_{1}-β_{32}∙μ_{2}$

$α_{31}=δ_{31}$

$α_{32}=δ_{32}-β_{32}∙δ_{21}$

$α_{33}=δ_{33}-β_{31}∙δ_{11}-β_{32}∙δ_{22}$

So, the parameters of PICS are just linear combinations of the parameters in PICS-R.
