# Supplementary material for: Estimation of Treatment Policy Estimands for Continuous Outcomes Using Off‐Treatment Sequential Multiple Imputation
Source: Pharm Stat. 2024 Aug 4;23(6):1144–55. doi: 10.1002/pst.2411 (PMC11602932; doi:10.1002/pst.2411)
Supplement: Supplementary file 6 [file PST-23-1144-s002.docx]

Appendix 2: Data Generation Model

# 1 Expected values of the treatment policy

For 10% Discontinuation (5%, 3%, 2%):

$\begin{matrix} E[Y_{0}]=μ_{0}^{on} \\ E[Y_{1}]={0.95∙μ}_{1}^{on}+{0.05∙μ}_{1}^{off} \\ E[Y_{2}]={0.92∙μ}_{2}^{on}+{0.08∙μ}_{2}^{off} \\ E[Y_{3}]={0.90∙μ}_{3}^{on}+{0.10∙μ}_{3}^{off} \end{matrix}$

For 20% Discontinuation (10%, 6%, 4%):

$\begin{matrix} E[Y_{0}]=μ_{0}^{on} \\ E[Y_{1}]={0.90∙μ}_{1}^{on}+{0.10∙μ}_{1}^{off} \\ E[Y_{2}]={0.84∙μ}_{2}^{on}+{0.16∙μ}_{2}^{off} \\ E[Y_{3}]={0.80∙μ}_{3}^{on}+{0.20∙μ}_{3}^{off} \end{matrix}$

For 50% Discontinuation (25%, 15%, 10%):

$\begin{matrix} E[Y_{0}]=μ_{0}^{on} \\ E[Y_{1}]={0.75∙μ}_{1}^{on}+{0.25∙μ}_{1}^{off} \\ E[Y_{2}]={0.60∙μ}_{2}^{on}+{0.40∙μ}_{2}^{off} \\ E[Y_{3}]={0.50∙μ}_{3}^{on}+{0.50∙μ}_{3}^{off} \end{matrix}$

The exact values of $μ_{j}^{on}$ and $μ_{j}^{off}$ depend on the timepoint and off-treatment scenario.

# 2 Details of the DGM

## 2.1 Stage 1 - Generating on-treatment and off-treatment Data

All multivariate normal on treatment outcomes are simulated based on the mean vector and variance-covariance matrix for the on-treatment control arm and then adjusted by the active treatment effect if the subject is in the Active group and a subject specific on-treatment heterogeneity is applied to each simulated value to add a small amount of patient specific noise. 

To generate the set of off treatment values, on-treatment values for the second timepoint onwards are adjusted according to the expected value for the off-treatment scenario and subject specific heterogeneity. For “return to baseline” scenarios the off-treatment values are adjusted to make the expected value equal to the baseline expected value for each treatment. In the “same as active” scenarios the off-treatment values are adjusted so that the expected value matches that of the Active, on treatment value for the same time point.

Let ${\boldsymbol{Y}}_{C}=(Y_{0C}, Y_{1C}, Y_{2C}, Y_{3C})'$ be the normal outcome vector for a control treatment with mean ${\boldsymbol{μ}}_{C}$ and covariance matrix ${\boldsymbol{Σ}}_{C}$  i.e.  ${\boldsymbol{Y}}_{C}\sim MVN({\boldsymbol{μ}}_{C}, {\boldsymbol{Σ}}_{C}).$ 

Let $\mathbf{Δ}={(Δ_{0},Δ_{1},Δ_{2}, Δ_{3})}^{'}$ be the vector of expected differences for active treatment compared with control.

Also define $u \sim normal(0,θ_{on}^{2})$ and $v \sim normal(0,θ_{off}^{2})$ be the patient specific heterogeneity for on and off treatment outcomes respectively.

The patient on-treatment outcome vectors for group Z can be created as:

${\boldsymbol{Y}}_{Z}^{on}={\boldsymbol{Y}}_{C}+ I_{[Z=A]}∙\boldsymbol{Δ} +{\mathbf{1}}_{4}∙u$

The patient off-treatment outcome vectors for group Z in the **return to baseline** scenarios can be created as: 

${\boldsymbol{Y}}_{Z}^{off}={\boldsymbol{Y}}_{Z,-1}^{on}-{\boldsymbol{μ}}_{C,-1 }-I_{[Z=A]}⋅ {\mathbf{Δ}}_{-1}+{\mathbf{1}}_{3}⋅μ_{0C}+ {\mathbf{1}}_{3}∙v$

The patient off-treatment outcome vectors for group Z in the **same as active** scenarios can be created as:

${\boldsymbol{Y}}_{Z}^{off}={\boldsymbol{Y}}_{Z,-1}^{on}+I_{[Z=C]}⋅ {\mathbf{Δ}}_{-1}+{\mathbf{1}}_{3}∙v$

Where:

- $Ι_{[∙]}$ is an indicator function taking the value 1 when condition $[⋅]$ is true and zero otherwise.

- ${\boldsymbol{Y}}_{Z, -1}^{on}$ is the vector of on-treatment outcomes for a patient in group Z with the first element removed.

- ${\boldsymbol{μ}}_{C, -1}$ is the vector of expected values of the control treatment with the first element removed.

- ${\mathbf{Δ}}_{-1}$ is the vector of expected values of active treatment effects with the first element removed.

- $μ_{0C}$ is the expected value of the control treatment at timepoint 0 (baseline).

- ${\mathbf{1}}_{\boldsymbol{n}}$ is an n-dimensional vector with each element being the number 1.

The multivariate normal distribution used to simulate ${\boldsymbol{Y}}_{C}\sim MVN({\mathbf{μ}}_{C}, {\mathbf{Σ}}_{C})$ was:

${\boldsymbol{Y}}_{C}\sim MVN([\begin{matrix} 2.14 \\ 2.47 \\ 2.52 \\ 2.54 \end{matrix}], [\begin{matrix} 0.45 & 0.46 & 0.46 & 0.47 \\ 0.46 & 0.66 & 0.62 & 0.63 \\ 0.46 & 0.62 & 0.65 & 0.63 \\ 0.47 & 0.63 & 0.63 & 0.68 \end{matrix}])$

And the active treatment effects $\mathbf{Δ}$ were:

$\mathbf{Δ}= {[\begin{matrix} 0 & 0.1 & 0.1 & 0.1 \end{matrix}]}^{'}$

The on and off-treatment heterogeneity standard deviations $θ_{on}$ and $θ_{off}$ were both set to 0.3.

This creates a single set of On-treatment and Off-treatment values for each subject and each scenario and is based on a single draw from the multivariate normal distribution. This allows the off-treatment values to be the same outcomes generated for the on treatment but adjusted for the scenario of interest.  

## 2.2 Stage 2 - Selecting patients to discontinue from treatment

Once both sets of On and Off treatment outcomes values have been generated, a discontinuation process is applied to the on-treatment data to determine if a subject discontinues. To flag the subjects that discontinue we used a heuristic selection process based on a propensity score.

For the $j$-th timepoint we defined a conditioning parameter $ω_{j}$ using the standard deviation $σ_{j}$ as:

$ω_{j}=\frac{0.5}{σ_{j}}$

We then use the conditioning parameter as the basis for a simple logistic selection model which for the DAR process is:

$P(D_{j}^{ DAR}=1\vert Y_{j-1})=logistic(ω_{j}∙Y_{j-1})$

And for the DNAR process:

$P(D_{j}^{ DNAR}=1\vert Y_{j})=logistic(ω_{j}∙Y_{j})$

To select the subject to discontinue we simulate $u\sim uniform(0,1)$, apply the inverse logistic transform (the logit) and subtract that value from both sides which creates a propensity like relative measure of the simulated on-treatment values. 

$\begin{matrix} κ_{j}^{DAR}=(ω_{j}∙y_{j-1})-logit(u) \\ κ_{j}^{DNAR}=(ω_{j}∙y_{j})-logit(u) \end{matrix}$

The measure $κ_{j}$ is then ranked and percentages below the required percentage threshold for the scenario are selected. This was to ensure exact proportions of subjects were selected for each simulated trial. 

The actual percentage thresholds for selection are based on the discontinuation rates for the particular scenario.

## 2.3 Stage 3 – Selecting patients to withdraw from the trial

Once patients have been selected as discontinuing treatment a simple MCAR selection process is applied for selecting the patients that withdraw from the trial. We simulate $u\sim uniform(0,1)$ and select the patients with values of $u$ below the threshold for the timepoint at which they discontinued. Similar to discontinuation selection, the actual percentage thresholds for withdrawal selection are based on the withdrawal balance for the particular scenario (e.g The 50% withdrawal scenario “More Late” would have thresholds of 20% at timepoint 1 and 80% at timepoints 2 or 3). If patients are selected to withdraw all subsequent values are set to missing, otherwise all their off-treatment values are kept.
