# Supplementary material for: Estimation of Treatment Policy Estimands for Continuous Outcomes Using Off‐Treatment Sequential Multiple Imputation
Source: Pharm Stat. 2024 Aug 4;23(6):1144–55. doi: 10.1002/pst.2411 (PMC11602932; doi:10.1002/pst.2411)
Supplement: Supplementary file 7 [file PST-23-1144-s005.docx]

Appendix 3: Bias and Variance Discussion

# 1 Comparison of bias between off-treatment scenarios

To understand the bias for the treatment effects and group means for the “Return to Baseline” and “Same as Active” scenarios it is useful to consider a simplified setup of the final timepoint in isolation. For each treatment group $Z∈(C,A)$, we define:

- $n_{1Z}$ as the number of patients completing the study on-treatment and $μ_{1Z}$ as the expected change from baseline FEV_1_ for these patients.

- $n_{2Z}$ as the number of patients completing the study but discontinued treatment and therefore provide off-treatment data at the final timepoint. Also define $μ_{2Z}$ as the expected change from baseline from these patients.

- $n_{3Z}$ as the number of patients that discontinued treatment and then withdrew from the study creating missing data at the final timepoint.

In both “Return to Baseline” and “Same as Active” scenarios the missing data for patients that discontinued treatment were generated to be similar to the patients that discontinued treatment but completed the study (i.e. both would have the same expected value $μ_{2Z}$). Concentrating on the scenarios that also assumed 50% of patients discontinuing treatment would then withdraw from the study to create missing data (i.e. $n_{2Z}=n_{3Z}$),it means the expected change from baseline in FEV_1_ for the full treatment policy for each group Z would be:

$E[Y_{Z}^{Full}]=\frac{n_{1Z}}{n_{1Z}+{2n}_{2Z}}∙μ_{1Z}+\frac{2n_{2Z}}{n_{1Z}+{2n}_{2Z}}∙μ_{2Z}$

We use this expected value as a benchmark for comparing the relative bias in both off-treatment scenarios that would result from making a naïve “common MAR” assumption that the $n_{3Z}$ missing outcomes are expected to be an aggregate of all the observed on- and off-treatment outcomes.

Under the common MAR assumption, the expected value of change from baseline FEV1 would be the same as the expected value of the observed data which would be:

$E[Y_{Z}^{MAR}]=\frac{n_{1Z}}{n_{1Z}+n_{2Z}}∙μ_{1Z}+\frac{n_{2Z}}{n_{1Z}+n_{2Z}}∙μ_{2Z}$

The bias from making this assumption would then be,

$E[Y_{Z}^{MAR}]-E[Y_{Z}^{Full}]=\frac{n_{1Z}}{n_{1Z}+n_{2Z}}∙μ_{1Z}+\frac{n_{2Z}}{n_{1Z}+n_{2Z}}∙μ_{2Z}-\frac{n_{1Z}}{n_{1Z}+{2n}_{2Z}}∙μ_{1Z}-\frac{2n_{2Z}}{n_{1Z}+{2n}_{2Z}}∙μ_{2Z}$

And so,

$Bias_{Z}=(\frac{n_{1Z}}{n_{1Z}+n_{2Z}}-\frac{n_{1Z}}{n_{1Z}+{2n}_{2Z}})∙μ_{1Z}+(\frac{n_{2Z}}{n_{1Z}+n_{2Z}}-\frac{{2n}_{2Z}}{n_{1Z}+{2n}_{2Z}})∙μ_{2Z}$

From this expression we see that the bias for treatment group $Z$ is related to the proportion of patients observed to be on- and off-treatment and how different they are from the proportions of on- and off-treatment with respect to the full data. It also shows the bias is related to the expected values of the on- and off-treatment outcomes. This explains the rise in bias in the group means and treatment effects for both off-treatment scenarios when the discontinuation rate increased and created more missing outcomes (as we fixed withdrawal to 50% of treatment discontinuations). Specifically the increased missingness created a larger disparity between the proportions of observed on- and off-treatment data compared to the full proportions. 

Rearranging the expression further allows us to explain why the bias for the “Return to Baseline” scenario is larger than the “Same as Active” scenario for a given discontinuation rate, mechanism, and study withdrawal type. Simplifying the expression, the bias for treatment group $Z$ becomes:

$Bias_{Z}=\frac{n_{1Z}∙n_{2Z}∙μ_{1Z}-n_{1Z}∙n_{2Z}∙μ_{2Z}}{(n_{1Z}+n_{2Z})(n_{1Z}+{2n}_{2Z})}$

Using the on- and off-treatment expected values for the “Return to Baseline” and “Same as Active” scenario we see the bias as:

$Bias_{C}^{RTB}=\frac{400∙n_{1C}∙n_{2C}-0∙n_{1C}∙n_{2C}}{(n_{1C}+n_{2C})(n_{1C}+{2n}_{2C})}=\frac{400∙n_{1C}∙n_{2C}}{(n_{1C}+n_{2C})(n_{1C}+{2n}_{2C})}$

$Bias_{A}^{RTB}=\frac{500∙n_{1A}∙n_{2A}-0∙n_{1A}∙n_{2A}}{(n_{1A}+n_{2A})(n_{1A}+{2n}_{2A})}=\frac{500∙n_{1A}∙n_{2A}}{(n_{1A}+n_{2A})(n_{1A}+{2n}_{2A})}$

$Bias_{C}^{SAA}=\frac{400∙n_{1C}∙n_{2C}-500∙n_{1C}∙n_{2C}}{(n_{1C}+n_{2C})(n_{1C}+{2n}_{2C})}=\frac{-100∙n_{1C}∙n_{2C}}{(n_{1C}+n_{2C})(n_{1C}+{2n}_{2C})}$

$Bias_{A}^{SAA}=\frac{500∙n_{1A}∙n_{2A}-500∙n_{1A}∙n_{2A}}{(n_{1A}+n_{2A})(n_{1A}+{2n}_{2A})}=0$

Looking at the corresponding bias values for each group it can be seen that the “Return to Baseline” scenario will have more bias than the corresponding “Same as Active” bias for a given discontinuation rate. It is also possible to see there is no bias for the “Same as Active” active group because the expected values before and after treatment discontinuation are the same. 

Finally, we can also use these bias terms to understand the similar levels of bias seen in the treatment effects for both the “Return to Baseline” and “Same as Active” scenarios when they have equal treatment discontinuation rates and the same corresponding discontinuation mechanism, and study withdrawal type. Because equal patients were allocated to each group any scenarios with the same discontinuation rate, mechanism and withdrawal type have $n_{1C}=n_{1A}, n_{2C}=n_{2A}$ and $n_{3C}=n_{3A}$ (which we will denote, $n_{1}$, $n_{2}$ and $n_{3}$). In this setting the bias in the treatment effects for both scenarios have a shift of $\frac{100∙n_{1}∙n_{2}}{(n_{1}+n_{2})(n_{1}+{2n}_{2})}$.

# 2 The impact of missing off-treatment data on variability

To provide some theoretical guidance on the relationship between the amount of missing off-treatment data and the variance inflation, we consider a simplified scenario of a single final timepoint with three fixed groups of subjects within a given arm:

- Group 1 are those who complete the study on-treatment. Suppose there are $n_{1}$ subjects in this group. The observed average change from baseline in FEV1 at timepoint 3 is ${\bar{y}}_{1}$ with standard error $σ_{1}/\sqrt{n_{1}}$.

- Group 2 are those who complete the study, but experienced treatment discontinuation, so that they provide some data on-treatment and some data off-treatment. We assume there $n_{2}$ subjects in this group. The observed average change from baseline in FEV1 at timepoint 3 is ${\bar{y}}_{2}$ with S.E. $σ_{2}/\sqrt{n_{2}}$.

- Group 3 are those who also experienced treatment discontinuation but did not complete the study. This group provided some data on-treatment, discontinued from treatment, then (potentially) provided some data off-treatment and eventually withdrew from the study (perhaps at the same time they stopped treatment), and consequently have missing data. Assume there are $n_{3}$ subjects in this group. Their average change from baseline in FEV1 at timepoint 3 is not observed.

The total number of subjects is $n=n_{1}+n_{2}+n_{3}$.

Suppose for simplicity that $σ_{1}=σ_{2}=σ_{3}=σ$, assumed known.

Under the assumption that those subjects in Group3 are like those in Group 2, the estimated value of the change from baseline in FEV1 at timepoint 3 would be:

$\frac{n_{1}{\bar{y}}_{1}+(n_{2}+n_{3}){\bar{y}}_{2}}{n_{1}+n_{2}+n_{3}}$

Now, assuming independence between the means in groups 1 and 2, the variance of that estimator is 

$\frac{n_{1}^{2}}{{(n_{1}+n_{2}+n_{3})}^{2}}Var({\bar{y}}_{1})+\frac{{(n_{2}+n_{3})}^{2}}{{(n_{1}+n_{2}+n_{3})}^{2}}Var({\bar{y}}_{2})$

When we observe all the data there are $n_{2}+n_{3}$ subjects with on- and off-treatment data, and therefore $Var({\bar{y}}_{2})=\frac{σ^{2}}{n_{2}+n_{3}}$. But in the presence of missing data, $Var({\bar{y}}_{2})=\frac{σ^{2}}{n_{2}}$ because there are only $n_{2}$ subjects with on- and off-treatment data. 

When we observe all the data for all subjects, the above variance expression becomes:

$\frac{n_{1}^{2}}{{(n_{1}+n_{2}+n_{3})}^{2}}⋅\frac{σ^{2}}{n_{1}}+\frac{{(n_{2}+n_{3})}^{2}}{{(n_{1}+n_{2}+n_{3})}^{2}}⋅\frac{σ^{2}}{{(n}_{2}+n_{3})}=\frac{σ^{2}}{{(n_{1}+n_{2}+n_{3})}^{2}}⋅(n_{1}+n_{2}+n_{3})=\frac{σ^{2}}{n}$ 

When there are missing data, then the variance is: 

$\frac{n_{1}^{2}}{{(n_{1}+n_{2}+n_{3})}^{2}}⋅\frac{σ^{2}}{n_{1}}+\frac{{(n_{2}+n_{3})}^{2}}{{(n_{1}+n_{2}+n_{3})}^{2}}⋅\frac{σ^{2}}{n_{2}}=\frac{σ^{2}}{n^{2}}⋅(n_{1}+n_{2}{(1+\frac{n_{3}}{n_{2}})}^{2})$

The relative increase in the variance of the estimator in the presence of missing data would therefore be:

$\frac{\frac{σ^{2}}{n^{2}}⋅(n_{1}+n_{2}{(1+\frac{n_{3}}{n_{2}})}^{2})-\frac{σ^{2}}{n}}{\frac{σ^{2}}{n}}=\frac{1}{n}(n_{1}+n_{2}+2n_{3}+\frac{n_{3}^{2}}{n_{2}})-1$ $=\frac{1}{n}(n_{1}+n_{2}+n_{3}+n_{3}(1+\frac{n_{3}}{n_{2}}))-1=1+\frac{n_{3}}{n}(1+\frac{n_{3}}{n_{2}})-1=\frac{n_{3}}{n}(1+\frac{n_{3}}{n_{2}})$

The above expression says that the relative increase in the variance of the estimator due to missing data becomes larger as the proportion of subjects with missing data $\frac{n_{3}}{n}$ increases, but also that the impact of a certain proportion of subjects with missing data $\frac{n_{3}}{n}$ will be amplified if the number (proportion) of subjects with missing data $n_{3}$ ($n_{3}/n$) is larger than the number (proportion) of subjects with off-treatment data $n_{2}$ ($n_{2}/n$).  

Using the discontinuation and withdrawal scenarios from our simulation study, the increase in variance implied by this simplified set up would be:

- 10% Discontinuation and 50% Withdrawal 🡪 $0.05∙(1+\frac{0.05}{0.05})=10\%$ increase in variance for group means.

- 20% Discontinuation and 50% Withdrawal 🡪 $0.10∙(1+\frac{0.10}{0.10})=20\%$ increase in variance for the group means.

- 50% Discontinuation and 50% Withdrawal 🡪 $0.25∙(1+\frac{0.25}{0.25})=50\%$ increase in variance for the group means.

- 10% Discontinuation and 70% Withdrawal 🡪 $0.07∙(1+\frac{0.07}{0.03})=23\%$ increase in variance for group means.

- 20% Discontinuation and 70% Withdrawal 🡪 $0.14∙(1+\frac{0.14}{0.06})=67\%$ increase in variance for the group means.

- 50% Discontinuation and 70% Withdrawal 🡪 $0.35∙(1+\frac{0.35}{0.15})=116\%$ increase in variance for the group means.

Using these increases for the group means and making an independence assumption for comparing the groups, the variance inflation for the treatment effects in our discontinuation scenarios would be:

- 10% Control: 10% Active and 50% Withdrawal 🡪 10% Increase.

- 10% Control: 20% Active and 50% Withdrawal 🡪 15% Increase.

- 20% Control: 20% Active and 50% Withdrawal 🡪 20% Increase.

- 50% Control: 50% Active and 50% Withdrawal 🡪 50% Increase.

- 10% Control: 10% Active and 70% Withdrawal 🡪 10% Increase.

- 10% Control: 20% Active and 70% Withdrawal 🡪 45% Increase.

- 20% Control: 20% Active and 70% Withdrawal 🡪 67% Increase.

- 50% Control: 50% Active and 70% Withdrawal 🡪 116% Increase.

The increases in variance seen in our simulation study was less than the values in this simplified set up, with the maximum seen in the PIOS model which was a 27% and 83% increase compared to the full ANCOVA across the 50% and 70% withdrawal scenarios respectively. This suggests that using MI that conditions on previous values can be used to successfully reduce impact of missing off-treatment data on variance inflation.
